# Structural and hydrodynamic controls on fluid travel time distributions across fracture networks

**DOI:** 10.1073/pnas.2414901121

**Published:** 2024-11-14

**Authors:** Philippe Davy, Romain Le Goc, Caroline Darcel, Benoît Pinier, Jan-Olof Selroos, Tanguy Le Borgne

**Affiliations:** ^a^Fractory, Geosciences UMR 6118, Univ Rennes, CNRS, Rennes 35042, France; ^b^Fractory, Itasca consultants, Rennes 35042, France; ^c^Research and Post-Closure Safety, Swedish Nuclear Fuel and Waste Management Company (SKB), Solna SE-169 03, Sweden; ^d^Department of Sustainable Development, Environmental Science and Engineering, KTH Royal Institute of Technology, Stockholm SE-100 44, Sweden

**Keywords:** fracture networks, anomalous transport, DFN, hydrogeology

## Abstract

Fracture networks are preferential flow paths playing a critical role in a wide range of environmental and industrial problems. Their complex multiscale structure leads to broad distributions of fluid travel times, affecting many biogeochemical processes. Yet, the relationship between the fracture network structures, their hydrodynamic properties, and the resulting anomalous transport dynamics remains unclear. We use a large database of fracture network models to investigate the factors controlling fluid velocity and travel-time distributions across a wide range of networks, from synthetic to field-calibrated models, with aperture variability at both fracture and network scales. Analysis reveals that transport statistics have generic properties across investigated networks, including notably heavy-tailed travel time distributions. Networks of increasing complexity and heterogeneity lead to broader velocity distributions and more channeled velocity fields, where flow concentrates in a narrow channel web in the three-dimensional (3D) fracture structure. While heterogeneity in point-velocity statistics increases travel-time variability, channeling tends to reduce it. This counterintuitive phenomenon challenges current theories, which assume that long travel time power law exponents are determined solely by point-velocity statistics. By analyzing velocity and travel time statistics for different flow structures, we develop a coupled Continuous Time Random Walk framework capturing the unexpected control of the velocity field’s spatial structure on anomalous transport in fracture networks. This leads to a unique class of random walk models capturing the respective roles of velocity heterogeneity and spatial structure on transport in networks. These findings open a prospective for characterizing, modeling, and predicting transport dynamics in complex networks, with potential applications to geological, biological, and engineered networks.

## Significance

In fractured rocks, the complex flow path structures lead to a larger dispersion of dissolved chemicals than predicted by conventional transport models, with consequences on water resources, energy, and environmental processes. However, the nature and causes of such anomalous transport remain unclear. Here, we use the most extensive database of fracture network simulations to establish the controls and general properties of fluid travel time statistics. Complex networks lead to the emergence of flow channeling, where flow gets focused in a web of narrow channels. Contrary to expectations, we show that this attenuates anomalous transport. We thus present a class of random walk models that describe transport dynamics in complex networks, with potential generalization to geological, biological, and engineered networks.

Fractures are present in all geological material on Earth, where they constitute preferential flow paths for fluids and elements (1–4). The complex, heterogeneous, and multiscale structure of fracture networks leads to large hydrodynamic dispersion characterized by broad fluid travel time distributions. Such anomalous transport dynamics play a major role in driving contaminant transport (5), water chemistry (6), deep microbial communities (7), CO_2_ and hydrogen storage (8), energy extraction, (9) and rock weathering (10). Understanding the transport dynamics in complex networks is also a fundamental problem with direct applications in different contexts, including epidemic or rumor spreading (11–13), brain microvascular dysfunction (14), or traffic flow (15). Fractured rocks are examples of such complex systems, where dispersion originates from the heterogeneity of flow due to both a complex network made of a wide range of fracture sizes (2) enhanced by the variability of fracture apertures within fracture planes or between fractures (16).

Transport in heterogeneous media has been studied for several decades with the objective of linking the transport parameters to the heterogeneity structure of the media (3, 4). Basically, the problem can be seen as a particle visiting a distribution of local velocities, whose total travel time is the sum of successive time increments controlled by particle velocities and step sizes. When the time steps are independent, the problem is close to the one treated by the central limit theorem, which predicts that the distributions in cumulative time tend to *α*-stable forms (17). Among them is the normal (gaussian) distribution, which attracts all local distributions with finite variance. The normal distribution was the basis for the widely used Advection Dispersion Equation (ADE, e.g., ref. 18), hereafter referred to as the ADE or Fickian model. The latter assumes a constant macroscopic dispersion depending only on the mean velocity field and scale parameters. However, this model has been contradicted by many observations of transport heterogeneous media (e.g., refs. 3, 19, and 20), where dispersion was suggested to be non-Fickian.

Three main mechanisms have been identified for these anomalous transport dynamics (21–23): The first cause of anomalous transport, called the “Noah” effect (23, 24), is an abnormally high occurrence of very small velocities – or long local time increments. If the time increment distribution has a power-law tail with a density exponent larger than −3 (i.e., a tail exponent *α* of the *α*-stable distribution smaller than 2), the variance is infinite and the total residence time distribution has also a power-law tail with the same density exponent (25). As a particular case, if the density exponent is larger than −2, the average travel time is also infinite. This mechanism is the basis of non-Fickian transport frameworks such as the Continuous Time Random Walk (CTRW) model, broadly used for modeling transport in the subsurface (3). The second cause, called the “Joseph” effect (23), results from broadly distributed spatial steps, that may be associated to long-range correlations in particle velocities. In this case (e.g., ref. 26), the central limit theorem does not apply and the velocity and residence-time exponents differ (24). Lévy flights and Levy walks belong to this class of systems (27). In the context of geological systems, such long-range velocity correlation could be induced by the broad distribution of fracture sizes. The third cause, called the “Moses” effect (21), is due to nonstationarity in the velocity/transmissivity field, which entails an ergodicity breaking in velocities (e.g., the velocity distribution changes as a function of distance). This can be produced by several geologically relevant processes: nonstationary boundary conditions as when the particles are not introduced in proportion to the input flow as stationary conditions would require (e.g., refs. 28 and 29); a hierarchy of scales in the spatial distribution of permeability or transmissivity identified when comparing laboratory and field tests (30, 31) (see also the discussion in ref. 32); or temporal fluctuations (33).

Anomalous transport is the norm rather than the exception in fractured rocks, as shown by field tracer testing experiments (34–36) as well as numerical simulations (37–40) but why and how it emerges, and how it relates to the network structure and flow properties is still an open question. Since fractured rock are characterized by a broad distribution of velocities, of fracture sizes and a multiscale organization, all of the three effects discussed above may contribute to the observed anomalous transport behavior. Recent studies on transport in fracture networks have suggested that heavy-tailed travel time exponents can be directly linked to point-velocity statistics using the CTRW model (e.g., refs. 41–43). Yet, when considering different fracture network structures and aperture heterogeneities, this approach was found to overestimate the tailing of travel time distributions (38, 39). These findings indicate that the spatial structure of velocity—particularly its channeling properties—also influences anomalous transport exponents, beyond what can be explained by point statistics alone. As a result, it remains unclear how to effectively capture both velocity statistics and their spatial organization in a transport model that applies across different fracture network structures. Additionally, how these velocity statistics are influenced by the distribution of fracture hydraulic properties, sizes, and connectivity is still unknown. In porous media, percolation theory and critical path analysis have offered insights into how pore network structures affect flow and transport properties (e.g., refs. 44–47). To date, the applicability of these frameworks to describe transport dynamics in fracture networks remains an open question.

Prior to flow and transport modeling, geological fracturing is in itself a complex process which can result in a diversity of fracture structures and hydraulic properties with no consensus about a unified description (2, 48). Recent advances in this area have been obtained from Discrete Fracture Network (DFN) approaches, which represent the fractured media as a population of discrete fractures with orientation, size, transmissivity, and aperture distributions (49–51). Fractured systems are acknowledged as intricate multiscale networks, featuring fracture sizes ranging from millimeters to tens of kilometers. However, translating this observation into a statistical distribution remains a challenge [see the discussion in Bonnet et al. (2)]. The characteristics of open fractures (density, size, and orientation distributions) control network connectivity (52–55), while the bulk permeability and the organization of flow, including channeling, depends also on the transmissivity/aperture distribution in the fracture plane and in between fractures (56, 57).

Here, we present a large database of DFN simulations, covering synthetic, genetic, and field-calibrated models, to investigate the structural controls on transport dynamics in fractured rocks. Furthermore, for a given network structure, we investigate the effect of aperture fluctuations both at fracture scale and at network scale. We show that travel time statistics follow a generic distribution, whose analysis reveals the mechanisms driving transport dynamics across fracture networks. Networks of increasing complexities exhibit both broader velocity distributions and more channelized velocity fields. Strikingly, we find that the two properties have antagonist effects on travel time distributions, leading to a breakdown of established theories. We suggest a theoretical framework that captures both the effect of velocity distributions, induced by the network structures, and that of velocity correlation along particle trajectories, linked to flow channeling. These findings hence shed light on the mechanisms governing fluid travel times within fracture networks.

## Field Observations of Travel Time Distributions in Fracture Networks.

**Fig. 1.**
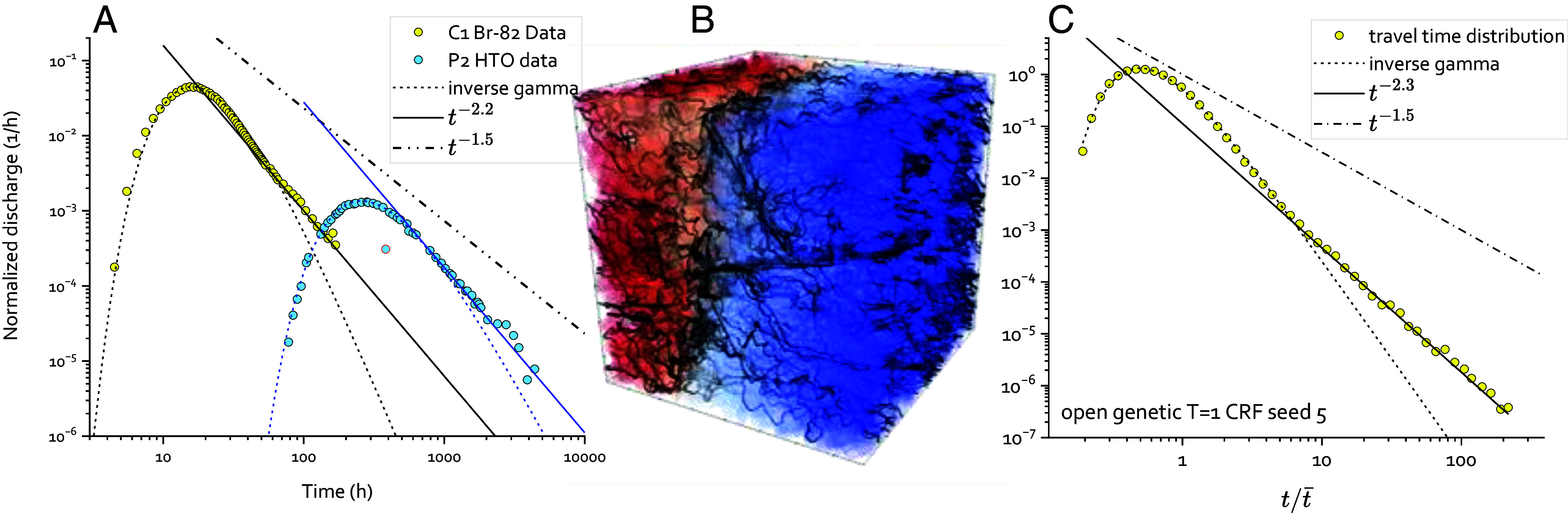
(*A*) Examples of BTC obtained from tracer test experiments carried out with nonsorbing tracers, ^82^Br and HTO, resp., at the Äspö Hard Rock Laboratory (Sweden) during the True Block Scale Test campaign (34). The solid lines are the power-law fits of the long-term trends; the dashed lines are the stretched inverse gamma fits for short and intermediate times (Eq. **1**). For comparison, the classical power law $t^{-3/2}$ representative of matrix diffusion processes is given in a dash-dotted line. (*B* and *C*) Illustration of a simulation run (open genetic DFN with variable aperture in the fracture plane, transmissivity structure T1&CRF in Table 1) with a visualization of particle paths on the DFN colored with hydraulic head values on a red-blue scale (*B*), and the resulting time distribution (*C*). As for field data, the gamma and power-law fits are indicated in solid and dashed lines, respectively, and the matrix diffusion scaling is shown as a dash-dotted line.

Travel time distributions can be measured in the field using cross-borehole tracer tests, an experiment in which a tracer is injected in a borehole and recovered at a different location where the breakthrough curve is measured in time (Fig. 1*A*). Such experiments have been performed in a broad range of geological contexts, systematically showing power law statistics of long travel times (e.g., refs. 58 and 59). The classical mechanism generally invoked to explain these long travel times is diffusion in the rock matrix, which leads to the power law $p(t)∼t^{-3/2}$ (58). Yet, the −1.5 scaling is generally not observed in field data that report power law evolutions with different exponents $p\left(t\right)∼t^{-a}$ (36, 60). In Fig. 1*A*, we show field tracer test data measured in one of the most instrumented fractured rock sites, the Äspö Hard Rock Laboratory in Sweden (34). This dataset covers the largest range of time scales measured for tracer tests in fractured rocks, from 1 h to almost one year. The general shape of a breakthrough curve (BTC) is a spread-out peak after the first arrival time, followed by a long tail in a log–log plot, showing a power-law trend. The latter significantly differs from the power law trend expected for matrix diffusion (Fig. 1*A*) suggesting the important role of advective dispersion processes, even over yearly time scales. As discussed in the following, the shape of field BTCs is very similar as those observed in our fracture network simulations (Fig. 1 *B* and *C*) that cover a broad range of fracture structures. Hence, although such simulations are necessarily simplified compared to the reality, they offer a relevant database to understand the key mechanisms driving long travel time statistics observed in the field.

## Assumptions and Database of DFN Models.

**Fig. 2.**
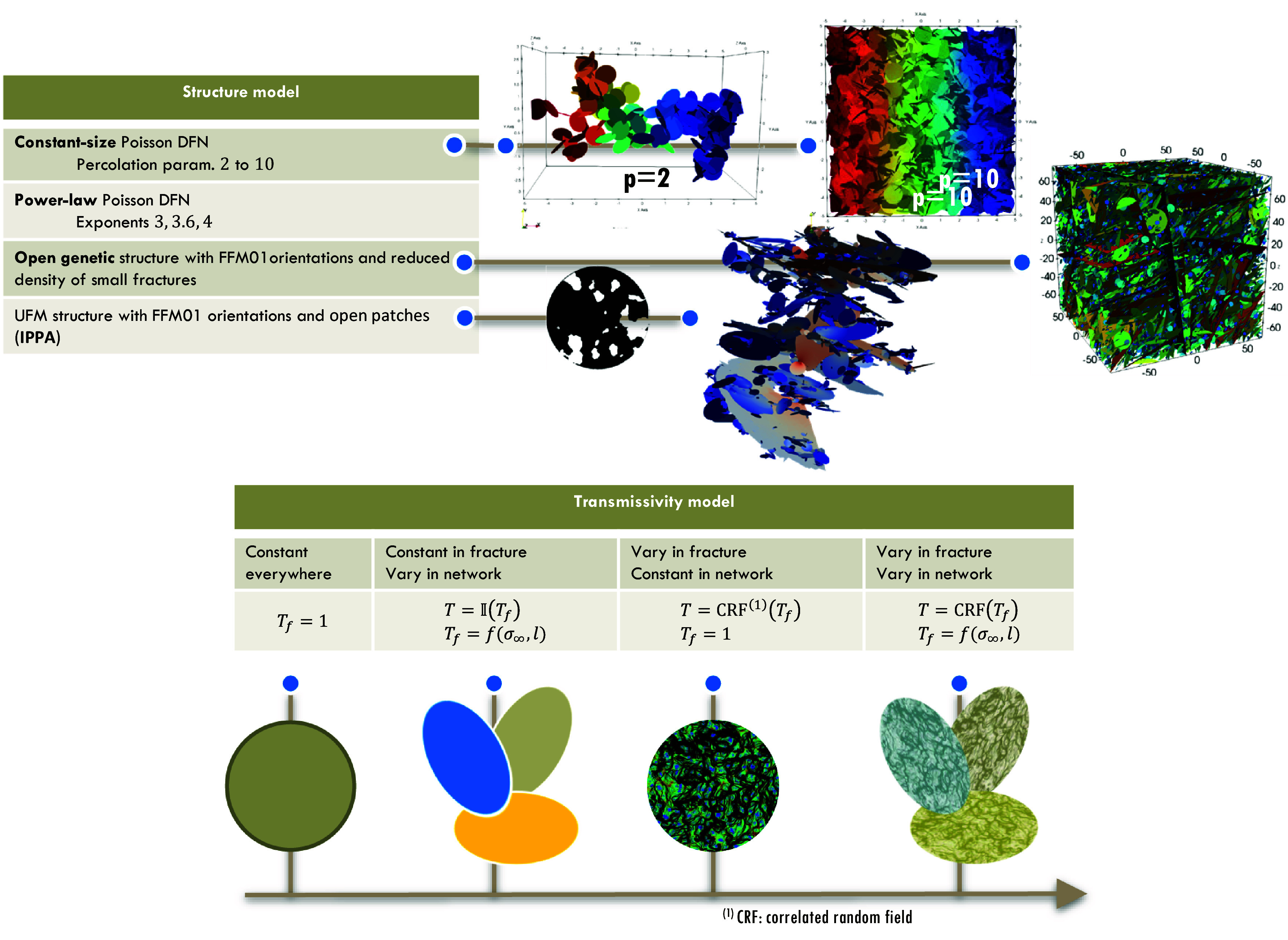
Examples of realizations for the simulated open-DFN structures (above), and for the four considered transmissivity models (below). We investigated the effect of structural heterogeneity by considering different structures for a constant transmissivity (Table 1). We then tested the effect of hydraulic heterogeneity for the open genetic structure, which represents a baseline structure model validated with extensive field data (32).

We approached the question of the relationship between DFN structures and transfer time distribution with a two-dimensional approach to complexity (Table 1): structural complexity (number and organization of fractures) on the one hand, and transmissivity variability (aperture fluctuations both at fracture scale and at network scale) on the other hand. The models studied explore these two complexity axes, with the central model being the genetic DFN, the validity of which was established with extensive field data (32). We thus use a large model database including networks of increasing complexities (Table 1 and Fig. 2), from generic networks with constant or power-law fracture size distributions (41, 56) to genetic models calibrated to field data (32). We consider three main fracture network topologies (Table 1): Poissonian, open genetic, and In-Plane PAtches (IPPA).

**Table 1.** Table of DFN structure (columns) and permeability (rows) models

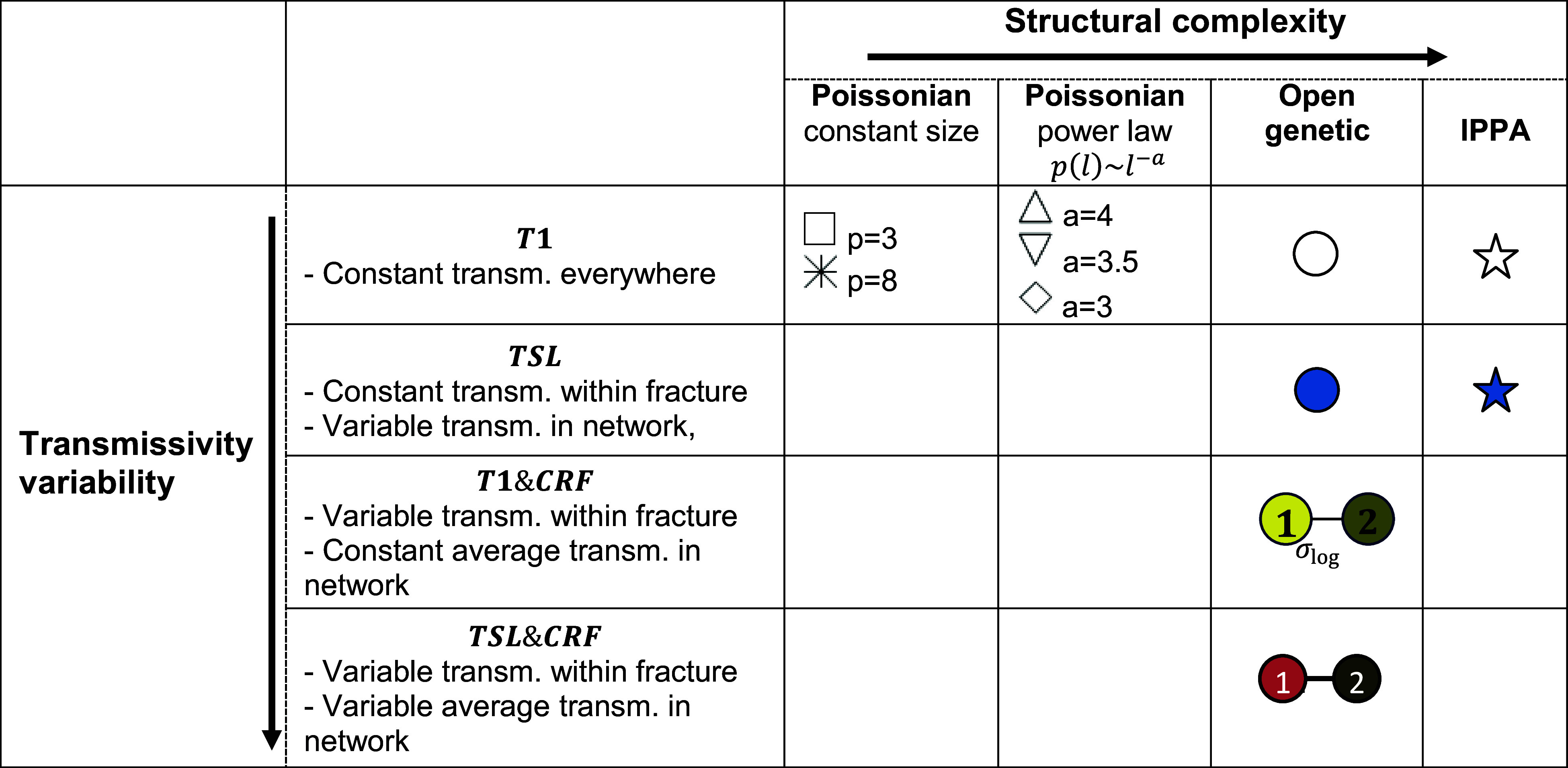

The shape of the symbol indicates the open DFN structure model. The color indicates the transmissivity model: fracture transmissivity exponentially decreasing with normal stress and linearly increasing with fracture size (*TSL*, blue), lognormally distributed within fracture planes with constant mean transmissivity and SD $\sigma_{\text{log}T}=1$ (*T*1*&CRF* yellow) and $\sigma_{\text{log}T}=2$ (*T*1*&CRF* light brown), lognormally distributed within fracture planes with variable mean transmissivity (according to *TSL*) and SD $\sigma_{\text{log}T}=1$ (*TSL&CRF* red) and $\sigma_{\text{log}T}=2$ (*TSL&CRF* dark brown). For constant fracture sizes, two different densities are simulated with a percolation parameter of three (square) and eight (double cross symbol). The DFN structures with power-law size distribution are generated with a power-law exponent of −4 (up triangle), −3.5 (down triangle), and −3 (diamond). The list of simulations is given in *SI Appendix*, Table S2.

In the Poissonian models, fractures are randomly positioned in the 3D domain. We first consider constant size fracture networks. Although these idealized models are very simple and far from the reality, they can generate very heterogeneous velocity and travel time distributions. This occurs particularly close to the percolation threshold, at low fracture densities, where connected flow paths become fractal (61). We then consider power-law fracture size distributions with Poisson generation and uniform fracture orientation distribution. Power-law distributions acknowledge the widespread observation that fracture sizes typically span several orders of magnitudes (2, 62). Such generic models have been broadly used to investigate basic properties of fracture network and thus constitute a reference (e.g., refs. 38, 41, 63, and 64).

The second type of fracture network topology is called open genetic, in which DFNs are generated from genetic rules of fracture nucleation growth and arrest (62). The fracture size distribution, which is an emerging property of these genetic rules, is a double power law with a small-scale exponent related to the size dependence of the fracture growth rate [an equivalent of Charles’ law (65)] and a universal large-scale exponent that depends on the arrest rules (see also ref. 66). This genetic DFN model is consistent with statistics derived from fracture network mapping (62) and has been shown to reproduce key flow characteristics in relevant geologic contexts (32). Here, we use genetic models calibrated to the Forsmark site in Sweden (67), likely the most characterized fractured rock site. Field data (*SI Appendix*, section S1) were collected and managed by the Swedish Nuclear Fuel and Waste Management Company (Svensk Kärnbränslehantering AB). The structure of these realistic DFN models rely on an initial description of the DFN geometrical properties (fracture size, aperture, transmissivity, orientation, shape, and spatial distributions), named geo-DFN model. As very commonly observed in the field, fractures are fully or partly sealed, and conversely open. In Forsmark, for example, the open fraction, defined as the cumulative area of fracture open surface divided by the total fracture area, is estimated to be between 15 and 25% (68, 69). The combination of the geo-DFN model and the open fraction defines the open DFN model (i.e., the DFN model once all the sealed parts are removed, leaving only the open parts). The open-DFN structure is the main determinant of the DFN connectivity. In the DFN model database, two models of open fraction were used. In the “open genetic” model, some fractures were fully open while others were sealed depending on the fracture size, in order to ensure flow connectivity similar to that measured at Forsmark (32).

The third type of fracture network topology is called IPPA, where fractures were partially sealed with open patches distributed within the fracture planes (32), representing contacts or filled areas. The fracture network generation is based on the open genetic model. Open patches in fracture planes are then generated by thresholding a correlated random field (*CRF*)—sealed if greater than the threshold, otherwise open—to a level such that the percentage of open area satisfies to the prescribed ***f**_op_*. The details of the patch generation are given in *SI Appendix*, section S2.

Transmissivity models are then assigned to the open fractures. The transmissivity models start from very basic, with constant transmissivity everywhere (transmissivity model *T* = 1, thereafter called *T*1). Then, following Follin and Stigsson (70), a second transmissivity model captures the observed positive correlation between transmissivity, normal stress acting on the fracture, and fracture size [*SI Appendix*, section S2 and ref. 32 for the detailed implementation]. It is noted *TSL* in the following with a fracture transmissivity exponentially decreasing with normal stress and linearly increasing with fracture size, consistently with field observations (35, 65). A third transmissivity model introduces in-plane variations of transmissivity based on a *CRF* spatial distribution with a lognormal variability of 1 and 2. The in-plane geometric mean is either the same in all fractures (*T*1) or distributed as in the *TSL* model. These transmissivity models are called *T*1 & *CRF* and *TSL* & *CRF*, respectively. Finally, we use the conventional assumption that relates fracture transmissivity and transport aperture by the classical cubic law (71). The DFN models are listed in Table 1 and illustrated in Fig. 2.

Flow and transport calculations were performed for all networks using the DFN.lab software developed by our team (https://fractorylab.org/dfnlab-software/). We computed the travel time distributions from particle tracking (72). We considered advective travel time distribution that characterize the fluid residence times in the fracture network and thus excluded diffusion into the rock matrix. The effect of matrix diffusion on travel time distributions has been extensively studied and shown to lead to a power-law trend at large travel times with an exponent of −1.5 (e.g., ref. 58). Yet this exponent is generally not observed in field tracer tests (Fig. 1*A*), suggesting that heterogeneous advection and structural heterogeneities play a major role on travel time statistics. Hence, including matrix diffusion would hide other important effects controlled by the fracture network structures. Following the broadly used assumption in DFN modeling (73), we assume here complete mixing in the fracture thickness as it is not possible to resolve flow in the fracture thickness when running simulations in networks of hundreds of thousands of fractures. This assumption is consistent with the range of time scale considered. For typical solute diffusion coefficients *D* and fracture apertures *α*, the diffusion time $\tau =a^{2}/D$ in the fracture thickness is on the other of seconds to minutes while the considered transport time scales range from days to years (Fig. 1*A*). Note however, that complete mixing is assumed only in the fracture thickness, not transversely. Hence, the redistribution of transported particles at intersections is explicitly resolved by tracking the partitioning of streamlines in different fractures. We used a flux weighted injection of particles to ensure the stationarity of the particle velocity distributions. Note that different injection modes may be used, such as resident injection, where particles are injected uniformly at the inlet independently of the local flow rate. Nevertheless, such injection modes introduce a long transient phase where the velocity statistics of particles evolves from the initial distribution to the asymptotic flux weighted distribution (38). This transient phase would potentially obscure the characterization of the relationship between transfer time and flow structure.

To investigate the variability of transport dynamics for a given structure, some models were generated several times with the same statistics but different seeds (*SI Appendix*, Table S2 and section S2). To characterize the structure of the DFN models and the corresponding flow networks, we used two dimensionless properties. The first is the percolation parameter *p*, which controls the network connectivity. The existence of a connected network across the domain is statistically obtained for *p* greater than a threshold *p_c_* that depends on fracture shape and orientation (54, 74). For uniformly distributed orientations and disk fractures, the percolation threshold is *p_c_* = 2.7 regardless of the fracture size distribution (53, 54). We have calculated the percolation threshold for all orientation distributions in the model database and found approximately the same value. The percolation parameter *p* is given in *SI Appendix*, Table S2 and section S2. It is much larger than the percolation threshold, i.e., *p* ≫ *p_c_*, for most of the simulations except for the first (*p* = 3). For such relatively densely connected networks, based on field observations as discussed above, a large number of independent pathways connect the input and output sides and the connected backbone is not fractal, contrary to networks at the percolation threshold *p* ≈ *p*_c_, (e.g., ref. 45). The second indicator is the ratio, $f=p_{32}/d_{q}$, between the total fracture surface *p*_32_ and the one occupied by the flow *d_q_* (*SI Appendix*, section S2 and Table S2). *d_q_* is equal to *p*_32_ when the flow is uniform; it decreases with flow heterogeneity (56, 57). The ratio *f* is an indicator of flow localization and channeling. The larger *f* is, the stronger is the flow localization. In the limit of constant flow in the network, *f* = 1. *SI Appendix*, Fig. S1*D* and section S4 shows *f* for the different runs. Channeling increases with the complexity of the DFN structure (IPPA > open genetic > power law > constant size), and with the variability of the transmissivity model (*TSL*&*CRF* > *TSL* > *T1*&*CRF* > *T*1). The variability from one realization to another within a model (same percolation parameter, structure model, and transmissivity model) is quite small.

## Velocity Distributions.

We systematically compute the Eulerian and Lagrangian velocity distributions (*n_L_* and *n_E_*, respectively) from DFN realizations of the models listed in Table 1 (72). The Eulerian distribution is directly calculated from the DFN flow field while the Lagrangian distribution is computed along streamlines from advective particle tracking at fixed spatial steps. Results are plotted as probability density functions of the inverse velocities ($v^{-1}$), to emphasize small velocities that correspond to large particle time steps (*SI Appendix*, Fig. S1 *A* and *B* and section S4). The tail of these distributions for large values of $v^{-1}$ typically follows a power law trends that potentially controls the statistics of particle transport times according to the Noah hypothesis (3, 41). We compute the tail power law exponents (Eulerian $\delta_{E}$ and Lagrangian $\delta_{L}$) in the range where the power law trend is established, over the same velocity range for both distributions. As expected, the resulting exponents $\delta_{E}$ and $\delta_{L}$ display a significant correlation (*SI Appendix*, Fig. S1*C*).

The Eulerian velocity statistics generally become more heterogeneous (I.e. exponents become smaller) for increasingly complex and realistic networks (*SI Appendix*, Fig. S1*C*). The Lagrangian and Eulerian distributions are related because they both sample the same velocity structure. In the Lagrangian distribution, each velocity $v_{L}$ is weighted by the number of particles passing through the location, which is proportional to the flux (*q*), giving $\bar{q}n_{L}\left(v\right)=qn_{E}\left(v\right)$, where $\bar{q}$ is the mean value of *q*. The flux *q* is locally related to the velocity with *q* = *bv*, where *b* is the fracture aperture. For ideal sampling conditions and for constant apertures ($-\bar{q}=b-\bar{v}$), it follows that $n_{L}\left(v\right)∼\frac{v}{\bar{v}}n_{E}\left(v\right)$ (28), leading to $\delta_{L}=\delta_{E}+1$. This result is recovered for constant aperture networks (open symbols in Fig. 5*A* and *SI Appendix*, Fig. S1*C*). For variable aperture networks, simulations exhibit significant differences with this simple relationship as they are modulated by the flow-velocity relation, i.e., by the effect of the aperture distribution. For open genetic models, aperture fluctuations tend to reduce the heterogeneity of velocities sampled by particles, which is characterized by an increase in the power law exponents $\delta_{L}$ compared to the simple flux weighting prediction (colored disks above the dashed line in Fig. 5*A*). This could possibly occur by increasing the flow toward low velocity areas characterized by larger apertures. For IPPA modes, with strong heterogeneity in the fracture plane, aperture fluctuations tend on the opposite to increase the heterogeneity of velocities (colored stars below the dashed line in Fig. 5*A*). Similarly, this could be produced by reducing the flux toward low velocity zones located in smaller aperture fractures.

Note that, while we compute the Lagrangian velocity distribution with a large number of particles (ranging from 400,000 to 1,000,000), some extremely small velocity regions are not visited by advective particles. In 3D DFNs, these dead ends are generally created by clusters with a single connection to the main flow paths, where flow is induced only by the gradients along the connecting intersection (75). These gradients are very small, and the resulting fluid flow in dead ends is several orders of magnitude smaller than in the rest of the network. This is shown in *SI Appendix*, Fig. S1*A*, where the distribution of dead-end velocities *v* is visible for *v* < 10^−9^ (i.e., *v*^−1^ > 10^9^), where the velocity distribution deviates from the long-tail power law. The probability of particles to enter these dead ends is about six orders of magnitude smaller that the slowest velocity sampled by particles. While our simulation already covers six orders of magnitudes in travel time probability (Fig. 1*C*), these dead ends would thus start affecting travel times of much lower probability of occurrence.

## A Generic Model for Travel Time Distributions in Fracture Networks.

**Fig. 3.**
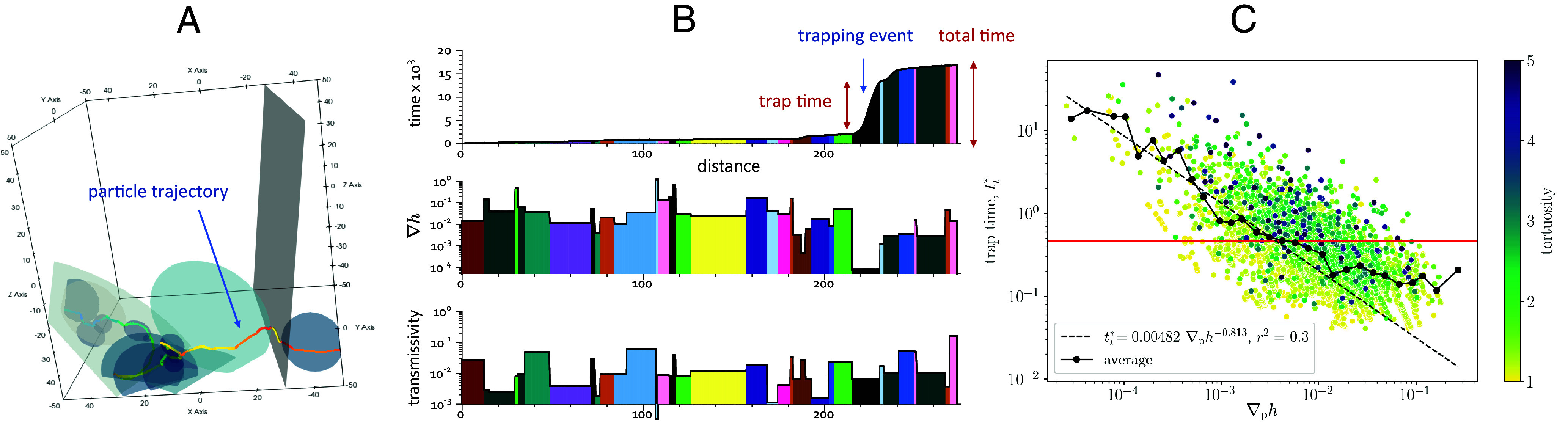
Illustration of the determinant of a particle travel time in an open genetic model with *TSL* transmissivity distribution. (*A*) 3D visualization of a particle path. The fractures in which the particle traveled are shown in transparency. (*B*) Analysis of a particle path in terms of cumulative travel time, head gradient ▽*h* between successive intersections, and local transmissivity *T*; each color corresponds to a fracture. In this trajectory, most of the particle travel time is spent in one fracture (in black) at a distance of about 220. The corresponding trap time represents a large fraction of the total time. (*C*) Plot of the trap time, defined as the longest time spent between two successive fracture intersections during the travel, as a function of the average head gradients along particle path between successive intersections. The color indicates the tortuosity corresponding to this trap time.

Breakthrough Curves (BTC) are obtained from distributions of particle travel times between injection and recovery locations (Fig. 3*A*). The shapes of simulated BTCs are similar as the field data (Fig. 1*A*), showing a spread-out peak after the first arrival time, followed by a long power-law trend (Fig. 1*C*). The corresponding power law exponents are similar as observed in the field (Fig. 1*A*) and significantly different from the −3/2 exponent expected for matrix diffusion. The strong similarity between field tests and numerical simulations does not directly validate the DFN model’s predictability for field BTCs. Nevertheless, this resemblance in the shapes of BTC and the long travel time exponents suggests that our extensive simulation database is relevant for explaining these properties, particularly in terms of the key structural and hydrodynamic factors controlling travel time distributions. The analysis of simulated travel time distributions suggests that, with a few exceptions and limits discussed hereafter, the BTCs can be described by the combination of a stretched inverse gamma (SIG) for early travel times and a power-law for the late travel times.

At short to intermediate times, the BTCs are often fitted by an IG function (28), which is considered as a good candidate to describe a long-tail power-law trend with a rollover at small time (76–78). To better represent the whole simulation database at short and intermediate times, we extend this IG model to a SIG with a power exponent parameter *s* in the exponential term:

[1] $$\mathrm{SIG}\left(t\right)=s\frac{\beta^{k-1}}{\Gamma \left(\frac{k-1}{s}\right)}t^{-k}\text{exp}\left(-{\left(\frac{\beta }{t}\right)}^{s}\right),$$

where Γ() is the gamma function, *k* and *s* are exponents, and β is related to the average travel time $\bar{t}$: $\beta =\bar{t}\frac{\Gamma \left(\frac{k-1}{s}\right)}{\Gamma \left(\frac{k}{s}\right)}$. Like the IG, the SIG function has a power-law tail $t^{-k}$ for large *t*. The shorter travel times, around the rollover, are controlled by the exponent *s* with a tighter distribution around the peak than IG when *s* > 1. For *s* = 1, the SIG is the standard IG function.

While it describes well the bulk of the BTC for both field and simulation data, the SIG does not describe the latest arrival times (Fig. 1 *A* and *C*), where a power-law trend different from the SIG tail is observed beyond a transition time *t_c_* with an exponent *α* lower than *k*:

[2] $$pw\left(t\right)∼\frac{a-1}{t_{c}}{\left(\frac{t}{t_{c}}\right)}^{-a}.$$

We thus identify a transition in the BTC at travel times (approximately equal to six times the mean in the example of Fig. 1*C*). that marks a transition between two regimes). The first part is well described by a SIG function and the second part by a power law decay whose exponent differs from the IG power law tail. This makes the travel time distributions more complex than usually assumed (76–78). The combination of the two functions is the model with the least number of parameters (five parameters) that ensures a good agreement with the simulated data. It describes the probability density function of travel times over six orders of magnitude in probabilities and three orders of magnitude in travel times (*SI Appendix*, section S6). The BTC model parameters are the average travel time $\bar{t}$, the transition time *t_C_* and three exponents hereafter called the stretched exponent (*s*), the gamma exponent (*k*), and the long-tail exponent (*a*). The parameters are calculated from a three-stage maximum likelihood procedure described in *SI Appendix*, section S6.

The gamma/power-law model generally applies to all models (see examples in Fig. 1*C* and *SI Appendix*, Figs. S2 *A* and *D* and S4 and sections S5–S7). The first moment $\bar{t}$ evolves linearly with distance (*SI Appendix*, Fig. S2 *B* and *E*). The evolution of the SD of travel time $\sigma_{t}$ ranges from Fickian $\sigma_{t}∼\surd x$ for the least heterogeneous structures (*SI Appendix*, Fig. S2*B*) to non-Fickian for most structures $\sigma_{t}∼x^{\beta }$ with $\frac{1}{2}<\beta <1$ (*SI Appendix*, Fig. S2*E*). The contribution of the long-tail power law to the second moment can be as much as 70% (*SI Appendix*, Fig. S6 and section S8). It thus exerts a strong control on the dispersion rate for all models that are sufficiently complex in structure or in transmissivity. Note that for the IPPA structures characterized by variable transmissivity (*TSL*), the travel time distributions exhibit a pronounced long-tailed power law distribution that dominates most of the travel time distribution. Hence, the exponents *k* and *a* are close to each other and the BTCs can be fully described by the SIG (*SI Appendix*, Fig. S4 and section S7).

## Noah, Joseph, and Moses Effects.

As discussed in the introduction, three main mechanisms can explain the observed power statistics of long travel times: the Noah effect (the abnormally high occurrence of very small velocities), the Joseph effect (the abnormally high occurrence of large numbers of steps), the Moses effect (the nonstationarity of the velocity field). Here, the Moses effect is minimized by the large domain sampled and the fact that particles are introduced proportionally to the flow, which leads to a stationary distribution of Lagrangian velocities.

To discriminate the dominant mechanism between the Noah and Joseph effects, we investigated the statistics of transport times, transmissivities, head gradients, and tortuosity along particle paths. Fig. 3*A* shows an example of particle trajectory in an open genetic model with TSL transmissivity distribution. Along this trajectory, the total travel time was dominated by one event at position *x* = 220 (Fig. 3*A*), corresponding to an extremely low velocity fracture. In this example, such low velocity was caused by an extremely low hydraulic head gradient ∇*h*, while the transmissivity *T* was close to average. This trapping effect is typical of the Noah mechanism. To test the generality of this effect, we computed the trap time, defined as largest time increment for transport across a fracture along the particle trajectory, for all trajectories. *SI Appendix*, Fig. S8*A* shows the trap time as a function of the total particle travel time, indicating a clear positive correlation between these two variables (*SI Appendix*, section S9). This analysis suggests the travel time of a given particle across the network is generally dominated by one extreme event characterized by the occurrence of a very low velocity at a specific fracture visited by this particle. The magnitude of trap times is mostly driven by the occurrence of abnormally small local hydraulic head gradients (Fig. 3*C*) and is poorly correlated other parameters such as the local transmissivity (*SI Appendix*, Fig. S8*D*) or the path tortuosity (*SI Appendix*, Fig. S8*C*). This suggests that among the different factors that could cause low velocities and large trap times (i.e., network topology, aperture variability between fractures, aperture variability within fracture planes), the network topology is the most important. While aperture variation can lead to locally small transmissivities, the network structure can connect areas of very similar pressure, leading to close to zero hydraulic head gradients applied on certain fractures.

The Joseph effect is not identified as playing an important role since travel times are dominated by the occurrence of a localized event along the particle trajectory and not by a property of the whole trajectory, such as tortuosity. Furthermore, the tortuosity of particle paths, and therefore the number of steps, varies in a relatively narrow range compared to the trap times (*SI Appendix*, Fig. S8*C*). We also note that most of the considered fracture networks are well above the percolation threshold, which implies that the structure of the flowing network is not fractal. This is confirmed by the linear evolution of the mean particle travel time with distance (*SI Appendix*, Fig. S2 *B* and *E* and section S5).

The dominant control of the Noah effect on travel time distributions observed here for fracture networks is similar as observed in other studies on transport in fracture networks (e.g., refs. 36 and 41) and in porous media (e.g., refs. 26 and 79–81). The broad occurrence of this mechanism in both porous and fractured media is linked to the existence of broadly distributed velocity distributions with sufficient probabilities for particles to encounter abnormally low velocities that dominate the travel time distributions.

The Noah effect has been successfully described by the CTRW framework (e.g., refs. 3 and 19). This theory explains the broad travel time statistics *p*(*t*) by the power law distribution of fluid velocities *p*(*v*) (*SI Appendix*, section S10). When velocity statistics follow a power law $p\left(v^{-1}\right)∼{\left(v^{-1}\right)}^{-\delta }$, as observed in our simulations (*SI Appendix*, Fig. S1*A*), this implies a=δ. For a≤3, power law statistics are stable upon summation since the sum is dominated by the occurrence of extreme events. This leads to power statistics of the total travel time, $p\left(t\right)∼t^{-a}$. For most fracture networks studied, the probability of transported particles to encounter an extreme event in terms of travel time is sufficiently large for the power law statistics to be independent on system size (*SI Appendix*, Fig. S2*D* and section S5). For all the DFNs expect one, the long-tail exponents *a* are less than three and thus stable, i.e., independent of the distance traveled by the particles (*SI Appendix*, Fig. S2*F*). This implies that the power law statistics of travel time distributions are independent on the system size. The only exception is the most homogeneous model with constant fracture size and high density (*p* = 8), which has a long tail BTC exponent a > 3, placing it within the conditions of the central limit theorem where BTCs tend toward Gaussian distributions (*SI Appendix*, Fig. S2*C*).

## Control of Velocity Field Heterogeneity and Structure on Fluid Travel Times.

To describe and model the Noah effect in fracture networks, we focus on the link between the velocity field properties and the travel time statistics. We first investigate the effect of single point velocity statistics. In the CTRW framework (*SI Appendix*, S10), the scaling of small velocities (or at large *v*^−1^) controls the long-time power-law regime of the BTC. The relevant velocity distribution is that sampled by transported particles at fixed spatial steps (28, 41, 42), i.e., $p\left(v\right)=n_{L}\left(v\right)$. The latter is also often assumed to follow a simple flux weighting of the Eulerian distribution (82), leading to $p\left(v\right)\approx vn_{E}\left(v\right)$. We thus test the two corresponding hypotheses for the long tail transport exponent:

[3] $$a=\delta_{E}+1,$$

[4] $$a=\delta_{L}.$$

**Fig. 4.**
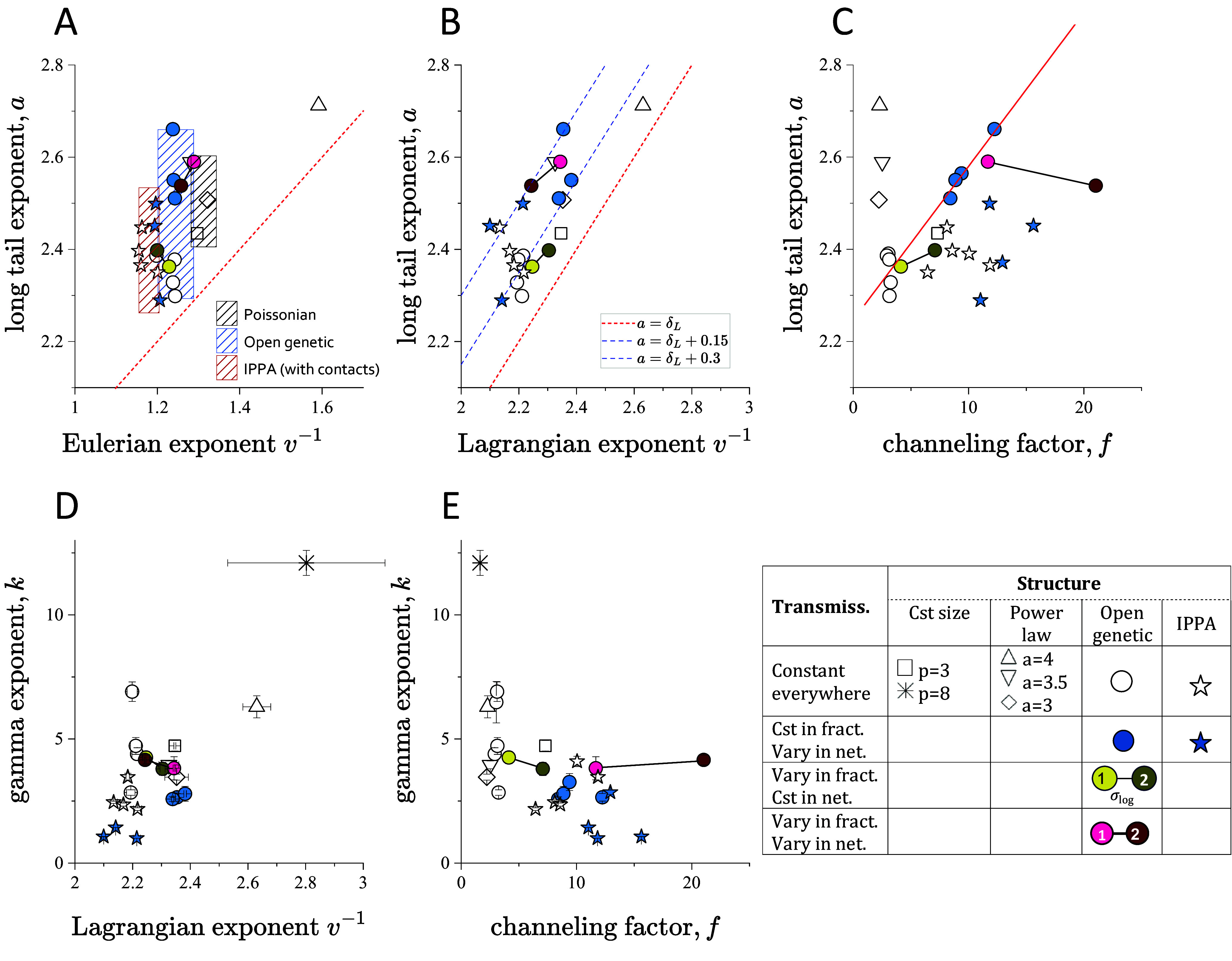
Evolution of the long-tail exponent *a* as a function of (*A*) the Eulerian velocity exponent of $v^{-1}\delta_{E}$, (*B*) the Lagrangian velocity exponent $\delta_{L}$, and (*C*) the channeling factor *f*. Gamma exponent *k* as a function of (*D*) the Lagrangian velocity distribution exponent $\delta_{L}$ and (*E*) the channeling factor *f*. The red dashed lines in panels *A* and *B* show the classical trends expected from Eqs. **3** and **4** respectively. The red line in panel *C* shows the trend $a=2.25+\frac{f-1}{25}$. The legend (*Bottom* row, *Right*) shows the DFN models. The hatched areas in *A* include the Poissonian models with both constant-size and power-law fracture sizes (black hatched), the open genetic models (blue hatched), and the IPPA models (red hatched), respectively. The pink and brown arrows indicate models with and without in-plane transmissivity variations.

The Fig. 4*A* shows the BTC long-tail exponent *a* as a function of the Eulerian $\delta_{E}$ exponents. Although there is a general positive correlation, it is relatively weak and different from the expected trend predicted by Eq. **3** with $a> >\delta_{E}+1$ for all simulations. This implies that transport is less anomalous than expected from a simple analysis of the Eulerian distribution, diverging from recent theoretical predictions (41). For a given fracture network topology (e.g., Poissonian, open genetic, IPPA), the Eulerian exponent $\delta_{E}$ does not vary much while the long travel time exponents are much more variable (Fig. 4*A*). The IPPA models have the smallest exponents between 1.15 and 1.20; open genetic structures have a slightly larger $\delta_{E}$ in the range of 1.2 to 1.3; and the Poissonian models (power-law and constant-size) models have an exponent $\delta_{E}$ around 1.3 to 1.32. The two outliers are the constant-size DFN with a high density of *p* = 8 ($\delta_{E}=1.8$) and the power-law DFN with a fracture size exponent of −4 ($\delta_{E}=1.6$). The latter model has a narrow fracture size distribution that makes it almost similar to the constant size models in terms of fracture connectivity (53). The introduction of aperture fluctuations in fracture planes did not significantly change the velocity statistics. For open genetic models, both constant aperture and variable aperture simulations remained within the blue shaded area in Fig. 4*A*, with less than 5% variability. When including locally closed patches within fracture planes, which represent contacts or filled areas (IPPA model), we observed a slight increase in velocity fluctuations (transition from blue shaded area to red shaded area in Fig. 4*A*). The long travel time exponents display a stronger correlation with the Lagrangian velocity exponents than with Eulerian exponents (Fig. 4*B*). However, they are still well above the prediction of the classical CTRW theory (Eq. **4** shown as a dashed red line in Fig. 4*B*). A dozen simulations are consistent with the relationship $a=\delta_{L}+0.15$, which includes most of the open genetic and Poissonian structures with constant fracture transmissivity. For the IPPA structure and for the runs with a variable transmissivity model (*TSL* and/or *CRF*), the long-tail exponents *a* are also larger than $\delta_{L}$ and appear to follow different trends (blue dashed lines in Fig. 4*B*).

The SIG exponents *k*, which quantifies the power-law trend for the first travel time regime (small to intermediate times), also follow a general positive correlation with the Lagrangian velocity exponents (Fig. 4*D*), although with a much larger slope. Hence, most of them are larger than three and thus not stable. For the most heterogeneous DFN, the SIG exponents are less than three and tend to be close to the late time travel time exponents *a*. In the limit where they are identical, the SIG describes the entire BTC. The SIG exponents show a negative correlation with the channeling factor (Fig. 4*E*), meaning that stronger channeling induces more dispersed travel times in this range of intermediate time scales.

We now investigate how the spatial structure of the velocity field affects the transport properties. For this we analyze the correlation of the long travel time exponents with the channeling factor *f*. The latter describes the formation high velocity channels that focus a large fraction of the flow. The increase in channeling is caused by three main sources of heterogeneity: 1) the DFN structure either (Poisson, open genetic, Ippa), 2) the variability in transmissivity from one fracture to another (*TSL*) and 3) the variability in transmissivity within the fracture plane (CRF). All these elements also display significant variability from one realization to another. Fig. 4*C* shows the evolution of the long travel time exponents as a function of the channeling factor *f*. The trend depends on the DFN structure (Fig. 4*C*). For the simplest model (power-law and constant-size DFN), *a* varies over a large range over a small range of channeling indicators *f*. For the most complex DFN structure (IPPA), large variations in f induce only small, if any, variations in a with a large scatter from one realization to another. For the open genetic models, the long-tail exponent increases approximately linearly with f ($a=2.25+\frac{f-1}{25}$) for all transmissivity models T1 and TSL. This trend is counterintuitive and opposite to the variations in the intermediate time exponent (Fig. 4*E*). It means that more channelized velocity fields lead to less anomalous transport. In-plane transmissivity variability (models T1-CRF and TSL-CRF) induces an increase in channeling but without significant changes in the long-time exponents (see brown and dark red disks compared to white and yellow disks in Fig. 4*C*). All together, these results indicate that channeling influences long-time travel time statistics in unexpected ways. In the following, we propose a framework allowing us to decipher the respective role of flow heterogeneity and structure in controlling long travel time statistics.

## Coupled CTRW model for transport in fracture networks.

The mismatch between the Lagrangian velocity and travel time exponents highlighted in Fig. 4*B* suggests a breakdown of the assumptions used in the classical CTRW framework. While this model was recently successfully used to model transport in one specific type of fracture network (41), it does not fully hold when considered across a broad range of network structures. Following the above analysis, we hypothesize that channeling may play a key role in this discrepancy.

The channeling structures of velocity fields in heterogeneous media implies that high velocities are organized in long and narrow channels while low velocities have more isotropic structures (54, 55). When considering velocity fluctuations along streamlines, the consequence is that large velocities tend to have a larger spatial correlation length than low velocities (26). This effect is generally absent in standard CTRW models where the step size is assumed to be constant and independent of the particle velocity (28, 41, 42). It can, in principle, be described by the correlated CTRW model that quantifies the full velocity transition probability of successive velocities experienced by particles. Thus, Kang et al. (39) used such spatial Markov process to define two velocity classes with distinct correlation properties—stronger correlations for the high-velocity class compared to the low-velocity class. They observed that this velocity-dependent correlation structure reduces anomalous transport compared to cases with velocity-independent transitions. However, the physical mechanism behind this process and its broader applicability remain unclear.

To understand and quantify the effect of flow channeling on anomalous transport, we introduce here a coupled CTRW model, in which the spatial step continuously depends on the particle’s velocity. Let us assume that the velocity correlation length, quantified by the step size ξ depends on the velocity as

[5] $$\xi ∼v^{\mu },$$

with μ > 0 the channeling exponent. It quantifies the possibility of particles to remain for a longer distance in a high-velocity channel than in a low-velocity zone. The travel time Δ*t* over a step size ξ is therefore:

[6] $$\Delta t\approx \frac{\xi }{v}∼v^{\mu -1}.$$

This leads to:

[7] $$p\left(\Delta t\right)=p\left(v\right)\frac{\mathrm{dv}}{d\Delta t}∼\Delta t^{\frac{\delta_{L}-\mu }{1-\mu }},$$

which differs from the temporal increment statistics assumed in current models (*SI Appendix*, section S10). Transport dynamics with coupled step size and velocity as proposed in Eq. **5** have been studied in the context of Levy walks (23). However, these theories have considered cases where μ < 0, where long travel times take longer steps than short travel times. For broadly distributed travel times, the latter assumption leads to broadly distributed step sizes and thus to a coupling of the Noah and Joseph effects discussed above. Here, the relation between step size and duration is $\xi {∼\Delta t}^{\frac{-\mu }{1-\mu }}$ with 0 < μ < 1, leading to a narrow distribution of step sizes according to Eq. **5**, so that the Joseph effect is absent.

The total travel time is the sum of the elementary time steps whose distribution is *p*(Δ*t*). For $\mu <\frac{\left(3-\delta_{L}\right)}{2}$, *p*(Δ*t*) is stable upon summation leading to the long tail exponent for $p\left(t\right)∼t^{-a}$ with,

[8] $$a=\frac{\delta_{L}-\mu }{1-\mu }.$$

Note that, contrary to the usual Levy Walk formulations, the step size distribution does influence the long tail exponent here because it is sufficiently narrow. We verified the validity of Eq. **8** with random walk simulations using a coupled CTRW algorithm

Using random walk simulations of both the coupled CTRW we have validated the predicted scaling heavy tailed scaling (Eq. **8**), confirming its stability with distance (*SI Appendix*, section S11). A consequence of Eq. **8** is that for μ > 0, $a> >\delta_{L}$. Hence, the flow channeling mechanism described by Eq. **5** tends to reduce anomalous transport. The comparison of the BTC simulated with the standard CTRW (curve in *SI Appendix*Fig. S9.b, section S11 with μ = 0) and the coupled CTRW model (curves in Fig. S11b with μ > 0) clearly illustrates the reduction of anomalous transport by flow channeling (Eq. 10 in *SI Appendix*, section S11), described here by increasing the value of the coupling exponent μ. When the channeling exponent μ is large enough, i.e., $\mu > >\frac{\left(3-\delta_{L}\right)}{2}$, this can even breakdown the stability of anomalous transport since *a* > 3, leading to a transition to Fickian transport (in *SI Appendix*, Fig. S9.b). The coupled CTRW model hence provides a general framework for understanding and quantify the effect of the velocity field’s spatial structure on anomalous transport dynamics through the coupling exponent μ. The qualitative similarity with the results of the two velocity-class spatial Markov model of Kang et al. (39) suggests a potential for relating the correlated CTRW and coupled CTRW models in a unified framework.

We hypothesize that the velocity-dependent correlation induced by channeling may explain the discrepancy betweent transport exponent *a* and the Lagrangian exponent $\delta_{L}$ predicted to be equal in the standard CTRW framework (41, 42). From Eq. **8**, we deduce:

[9] $$\mu =(a-\delta_{L})/(a-1).$$

**Fig. 5.**
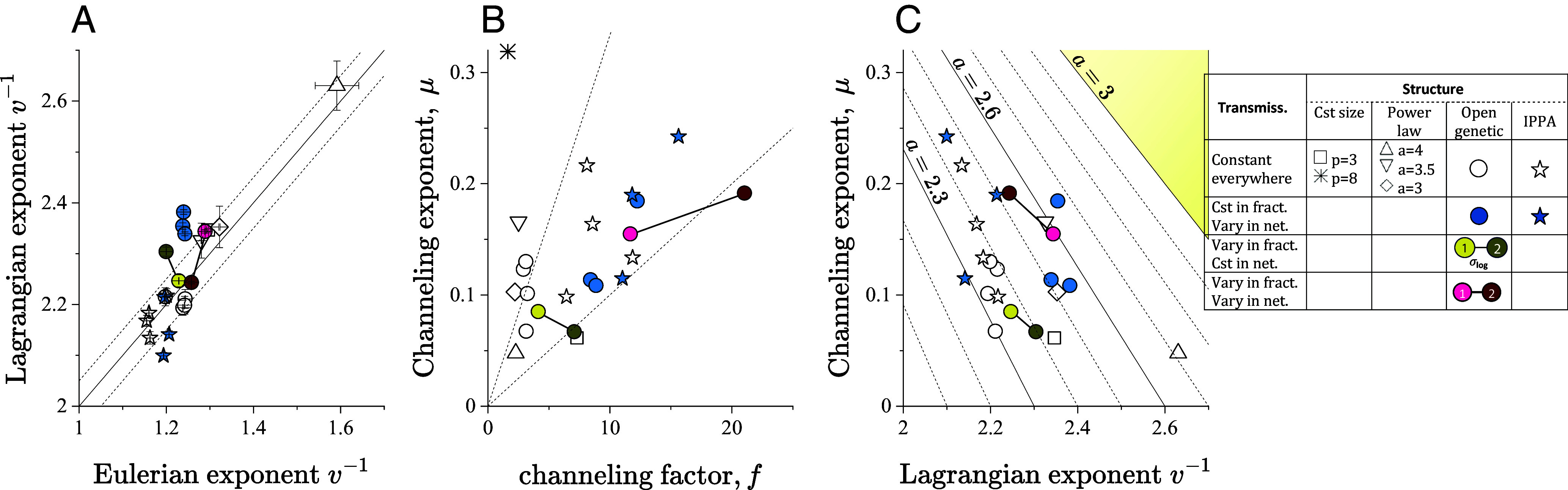
(*A*) the Lagrangian velocity exponent $\delta_{L}$ as a function of the Eulerian velocity exponent $\delta_{E}$ (dots) with the reference lines *y* = *x* + 1 (solid line) and *y* = *x* + 1 ± 0.05 (dashed lines). (*B* and *C*) Channeling exponent μ calculated from Eq. **9** as a function of the channeling intensity factor *f* (*B*) and the Lagrangian velocity exponent. (*C*) The dashed lines in *B* are *y* = 0.01*x* and *y* = 0.03*x*. The oblique lines in *C* are iso-values of the long-tail exponent *α* according to Eq. **9**; the yellow top right corner area corresponds to nonanomalous transport (*α* > 3). The DFN model symbols are the same as shown in the legend of Fig. 4.

In our series of simulations, μ varies between 0.05 and 0.3 and increases with channeling (Fig. 5 *B* and *C*). The rate of increase between μ and the channeling factor *f* depends on the transmissivity model (dashed lines in Fig. 5*B*). On average, models, it is greater for models with constant transmissivity (*T*1) than for those with transmissivity that varies across the network from a fracture to next (*TSL*, *T*1&*CRF*, *TSL*&*CRF*). The flow channeling model of Eq. **5** thus provides a mechanism for transport exponents to be larger than the velocity exponents (Figs. 4 *A* and *B* and 5*C*). This leads to less anomalous transport than expected from current anomalous transport theories.

## Discussion

In this study, we have used a large DFN model database to analyze the structural and hydrodynamic controls of fluid travel time distributions across a range of fracture networks. across the range of investigated fracture structures or transmissivity variabilities, travel time distributions exhibit a generic two-regime travel time distribution. For small to intermediate times, travel time distributions follow a SIG. At late times, we identify a transition between the SIG and a power law trend, whose exponent gives a large weight to very long travel times. Although it represents a small mass, the contribution of this long-tail power law on the dispersion is disproportionately large. The long-tail exponent can therefore be used as a metric to evaluate the anomalous nature of transport.

Analyzing the transport statistics along particle trajectories, we have shown that the observed anomalous transport dynamics are mostly driven by the Noah effect, i.e., the abnormally high occurrence of very small velocities. Hence, the travel time of a transported particle is generally dominated by one extreme event characterized by the occurrence of a very low velocity at a specific fracture visited by this particle. We have shown that these low velocities are mostly driven by the occurrence of low hydraulic head gradients and not so much by low fracture transmissivities. It remains an open question as to which properties of the DFN control the head distribution at the intersections, but studies on equivalent graphs suggest that the network topology exerts a strong control on it (83, 84).

The heterogeneity in fluid velocities increases with the complexity, and realism, of fracture networks. However, for a given network structure, there is surprisingly low variability in Eulerian velocity statistics (Fig. 4*A*), even when including fracture aperture fluctuations either at network or fracture scale. Consistently with the identified predominant role of hydraulic head gradients, Eulerian velocity statistics are mainly governed by the network topology, and are, to some extent, independent on the transmissivity model. This gives a special emphasis to the topology of the DFN with respect to other parameters (57, 85).

In contrast to recent findings that focused on a specific DFN structure (41), there is no clear link between the exponents characterizing the Eulerian velocity and travel time statistics when analyzing all networks together (Fig. 4*A*). We identified two mechanisms to explain this. The first mechanism is the sampling of the velocity field by transported particles. Aperture fluctuations alter the standard flux weighting law (28) (Fig. 5*A*) and can either give more weight to large velocities (open genetic models) or to low velocities (IPPA models). This significantly disperses the Lagrangian velocity exponent compared to the narrow range of Eulerian velocity exponents (Fig. 5*A*). The second mechanism is the channeling of the velocity field, which results in shorter distances spent by particles in low-velocity regions than in high-velocity channels, as quantified by Eq. **8**. The counterintuitive consequence is that channeling, i.e., the localization of large velocity areas in elongated channels, induces less anomalous transport for given point velocity statistics. This is quantified by the flow channeling exponent μ, which tends to decrease the impact of point velocity statistics on anomalous transport, as quantified by Eq. **8**. Although there is some scatter, this exponent shows a positive correlation with the channeling factor *f* (Fig. 5*B*), confirming the physical interpretation of this process.

These mechanisms are quantified by a coupled CTRW framework that captures the respective role of point velocity statistics, characterized by the Lagrangian velocity exponent, and flow structure, characterized by the channeling exponent (Fig. 5*C*). Networks with enhanced flow heterogeneity are generally more channelized due to flow conservation. Hence, the two mechanisms tend to compensate each other, leading to similar transport exponents for networks with different degrees of heterogeneity (Fig. 5*C*). Introducing aperture fluctuations tends to increase flow channeling (Fig. 5*B*), leading generally to less anomalous transport (Fig. 5*C*). This counterintuitive effect hence provides a mechanism controlling anomalous transport in fractured media. This conclusion may be dependent on the type of considered injection mode. For instance, a resident injection may lead to an oversampling of low velocity zones, which, in the case of highly channeled flows, may enhance anomalous transport.

These findings establish a general framework for linking flow heterogeneity and structure to transport dynamics across a wide range of fracture networks. They also highlight the need to investigate how fracture network structures control velocity field properties and trap formation driven by low hydraulic head gradients. The q-model originally developed to quantify the force fluctuations in random bead packs (86), and applied by some of us to brain microvascular networks could possibly explain why low head gradients form and how they relate to the network topology (87). Similarly, percolation theory and its extension, the Critical Path Analysis, may provide a rationale for the control of network structure and heterogeneity on velocity field properties, following the methodologies developed in the context of porous media (45, 47).

In summary, for most of the DFN consistent with field data, transport is anomalous in the sense that dispersion is controlled by the longest travel time, and the BTC results mostly from the “Noah” effect (abnormally high occurrence of very small velocities). Velocity correlation properties resulting from channeling lead to a breakdown of expected relationships between velocity and transport statistics. Surprisingly, this implies that channeling reduces the impact of low velocities on anomalous transport. This study has covered a range of models more than any other study (see refs. 1, 36, 38, 41, 56, and 88–92). Nevertheless, despite our caution to extract conclusions as general as possible, we do not exclude the possibility that specific DFN configurations may diverge from these generic dynamics. Note also that we have considered here fluid travel times estimated from advective transport simulations. In the future, these dynamics should be investigated with coupled advection–diffusion, hence opening opportunities to understand solute dispersion in fracture networks across different Peclet number regimes.

## Supplementary Material

Appendix 01 (PDF)

## Data Availability

The probability distributions of travel time, Eulerian and Lagrangian velocities for all calculated DFNs as well as the simulation data have been deposited in Gitlab (https://gitlab.com/fractorylab/saft-project) (72).
