# Supplementary material for: Structural and hydrodynamic controls on fluid travel time distributions across fracture networks
Source: Proc Natl Acad Sci U S A. 2024 Nov 14;121(47):e2414901121. doi: 10.1073/pnas.2414901121 (PMC11588132; doi:10.1073/pnas.2414901121)
Supplement: Supplementary file 1 — Appendix 01 (PDF) [file pnas.2414901121.sapp.pdf]

Supplementary information for “Structural and Hydrodynamic Controls on Fluid Travel Time Distributions Across Fracture Networks”

S1. Field case – the Forsmark site fracturing database

The realistic Discrete Fracture Network model used in this study was developed for the Formark site in Sweden, a crystalline rock formation selected by the Swedish government to host an underground repository for nuclear waste. The nuclear waste management company SKB has developed arguably the largest database in the world for fracture and flow. The database relies on more than 25 km of drilled boreholes, with highly detailed core mapping and video logging, flow testing, etc., in addition to surface detailed outcrop mapping and lineament mapping. This level of detail, particularly in terms of flow detection and spatial resolution, is rarely found. Many SKB reports describe the site properties (e.g., (1-4)) and the modeling efforts (5).

The site is divided into several fracture domains that differ in fracture density, orientation distribution, and geological parameters. The target domain for the future repository is named FFM01. It is moderately fractured with an average fracture density term of approximately 5 fractures per meter along boreholes and 5 main fracture sets (see Table S1).

Table S1. List of the different fracture sets for the FFM01 fracture domain at depths between -200 and -400 m with their intensity P_{32} and the Fisher parameters that define their orientation distributions. P_{32} values are taken from Table 6-33 in Glamheden*, et al.* (6) and Fisher parameters from Table C-1 in Follin (7).

Set name	$\boldsymbol{P_{32}}$ $(\mathbf{m^2/m^3})$	Fisher distribution parameters		
		Trend (°)	Plunge (°)	Kappa (-)
NS	1.292	292	1	17.8
NE	1.733	326	2	14.3
NW	0.948	60	6	12.9
EW	0.169	15	2	14.0
HZ	0.624	5	86	15.2

The intensity values (P_{32} as the surface of fracture per unit of volume and inferred from core logging and stereological analyses) in Table S1 correspond to the structure DFN model, i.e., including all the fractures, whether open, sealed or partly open. The open fraction f_{op} is measured from core logging by identifying open and partially open fractures, as an overall measure that does not indicate which fractures are open, either individually or in terms of their characteristics. At Forsmark it is estimated between 15 and 25% of the fracture network surface (2, 8).

Fracture transmissivity measurements based on Posiva Flow Logs (PFL) in the drilled boreholes are also available. PFL was originally developed by Posiva Oy to meet the demand for flow measurement techniques adapted to sparsely fractured rocks and low-permeability environments (9). Follin and Stigsson (10) analyzed the transmissivity of deformation zones at different depths and orientations. They conclude that the transmissivity is strongly dependent on the stress normal to the fault plane σ_n in an exponentially decreasing relationship $T_f \propto \exp(-\sigma_n/\ \sigma_c)$ where $\sigma_c = 4\ \mathrm{MPa}$. Fracture transmissivity is also assumed to vary with fracture size although this relationship is not supported by any data. Transmissivity models assume a power-law dependence, a relationship that accounts for variations in aperture with fracture size and the dependence of transmissivity on aperture (8, 11, 12). Analysis of the PFL flow logs in the boreholes led us to interpret the scaling of rock mass permeability as consistent with networks reaching the percolation threshold at a scale of ~10m, and fracture transmissivity increasing with fracture size (13). The reason why the percolation threshold is a scale and not a density as in the classical percolation theory (14) is the result of the power-law size distribution, which increases the probability of encountering larger and larger fractures as the system grows in size (15, 16).

All the above-mentioned data – from fracture orientation and intensity, open fraction and PFL testing- are successively incorporated in the definition of a DFN model for the FFM01 unit. The modelling steps include stereological analyses to infer 3D distribution of fracture properties from fracture intercepts along the well and outcrop mapping by stereology rules (17-19) and combination between outcrop mapping at different resolution scales. In this process, the distribution of fracture sizes is a key modelling issue, because of its important role in network connectivity, flow distribution and transport (15, 20-23). The DFN model(s) calibration and rejection steps resulting from the integration of the PFL data and used in the present study – the realistic open genetic and IPPA listed in the Table 1 of the main text – are referred in (13).

S2. DFN models

- **Numerical methods**

All the numerical simulations – from DFN generation to flow and transport calculations- are performed with the DFN.lab platform (https://fractorylab.org/dfnlab-software).

Each DFN sample is defined in a cubic box. Hydraulic boundary conditions are permeameter-like fixed constant heads on two opposite sides of the cubic box and no flow on the other four sides. The head difference between opposite sides generates the head gradient within the DFN and thus the flow.

A breakthrough curve is calculated by launching 400,000 to 1 million advective particles on the side with the largest head. The particles are introduced in the inlet border proportionally to the fracture flow, i.e., stationary injection, which removes a cause of non-ergodicity in the BTC structure (24-26). Note that diffusion in the fracture plane and in the matrix is not considered here as we focus on the control of flow heterogeneity and structure on transport dynamics. Table S2 of the Supplementary Information shows the list of runs used in this study.

- **Structure models**

The DFN structure models are described in detail below:

a. Constant-size: These are the simplest DFN structures with constant fracture sizes of $\boldsymbol{l}$ =1 m, randomly distributed in a cubic system of side $\boldsymbol{L}$ =10 m with orientations uniformly distributed. Even if several networks with different densities have been studied, we will focus on two end-member cases: a DFN with a percolation parameter p of 3, very close to the threshold ($\boldsymbol{p_c} = \mathbf{2.5}$) and a network with high density ($p = 8$) considered as homogeneous. As a reminder, the percolation parameter is written: $\boldsymbol{p} = \frac{\pi}{8}\sum_f \left(\frac{l_f}{L}\right)^3$, where $\boldsymbol{l_f}$ is the size of the fracture $\boldsymbol{f}$.

The three other models are considered as "realistic" realizations of the Forsmark site. The fracture networks have the characteristics of the FFM01 fracture domain, whose fracture orientations and intensities are described in Table 2. They all have quite a wide distribution of fracture sizes as it is in nature. The domain is a cube of 150 m side, and, unless otherwise specified, the size of the smallest fracture is 2 m and the largest may be up to the domain size.

- Power law: DFN with power-law fracture size distribution is common in modeling natural fracture networks(8, 27, 28) with a range of exponents of the density distribution that is likely varying between -3 and -4 (29). For this set of models, DFNs were computed with fracture centers following a Poisson process, i.e., a random distribution in space. 3 power-law exponents of -3.0, -3.5 and -4.0 were investigated.
- Open genetic: The third set of experiments was designed motivated by (i) more "mechanistic" simulations of the fracturing process, and (ii) the consideration that 80% of the total surface is clogged. The geo-DFN (i.e., with all fractures) is generated according to the so-called "genetic" model, as described in Davy, Le Goc, Darcel and Selroos (13), from fracture nucleation, growth and

arrest rules. Compared to Poissonian rules (i.e., fracture positions are independent of each other), the model produces a large percentage of T-intersections between fractures due to the arrest rule. To account for the distribution of fracture openness, the model assumes that fractures are either fully sealed or fully open, and the percentage of sealed fractures is a function of fracture size (see fig.8 in (13)). How the sealing varies with fracture size is a key parameter of the model connectivity; the one chosen is consistent with observations of the evolution of permeability as a function of scale(13). The surface density ratio between open-DFN and geo-DFN is 21%.

- Ippa: Based on the analysis that the open distribution is a critical unknown (e.g., (8)), we have built a new model set where each fracture has both open and sealed patches. The model set is called ippa for In-Plane PAtches. The open patches are generated by thresholding a self-affine correlated random field – sealed if greater than the threshold, otherwise open – to a level such that the percentage of open area satisfies to the prescribed $\boldsymbol{f_{op}}$. For the same connectivity reasons as the previous set of models (see previous section and (13)), only models where $\boldsymbol{f_{op}}$ increases with fracture size are sufficiently connected to reproduce the permeability scaling. Since the purpose of this paper is not to provide an exhaustive analysis of this model, we have chosen a set of parameters that are known to be compatible with flow observations(13): a correlation length of 20 m, a Hurst exponent of 0.8 for the self-affine correlations, $\boldsymbol{f_{op}}$ that grows with fracture size to the power of 0.4. The geo-DFN and the total open fraction are identical to the open-genetic model set (previous section).

- **Transmissivity models**

The DFN transmissivity models are:

1. **T1** - the transmissivity is constant, equal to 1, throughout the network.
2. **TSL** - for each fracture $\boldsymbol{f}$ the fracture transmissivity $\boldsymbol{T_f}$ is given by $\boldsymbol{f(\sigma_\infty, l)}$. It is constant by fracture but varies from fracture to fracture. $\boldsymbol{f(\sigma_\infty, l)}$ quantifies the fracture closure due to the remote stress tensor $\boldsymbol{\sigma_\infty}$ and the transmissivity-size dependence. The stress dependence is consistent with the analysis of Follin*, et al.* (8). As a very simple hypothesis, we assume that the transmissivity dependence on fracture size is linear. This is consistent with data (13), although slightly stronger than that used by Follin*, et al.* (8) ($\boldsymbol{T_f \propto l^{0.6}}$) or Hyman, Aldrich, Viswanathan, Makedonska and Karra (11) ($\boldsymbol{T_f \propto l^{0.5}}$). This gives the following expression:

$$f(\sigma_\infty, l) = l * \exp(-\sigma_n/\sigma_c)$$

where $\boldsymbol{\sigma_n}$ is the stress normal to the fracture plane and $\sigma_c = 4\ MPa$. The normal stress is calculated by projecting the remote stress tensor measured at a depth of 400m at the Forsmark site (6).

3. **T&CRF($\boldsymbol{s}$)**: $T_f(x) = T_f * CRF(x, s); T_f = 1$ - the transmissivity varies in the fracture plane $\boldsymbol{f}$ with a lognormal distribution of log standard deviation $\boldsymbol{s}$ and a spatial organization given by a Correlated Random Field (CRF). The transmissivity geometric mean per fracture is $\mathbf{T}$, regardless of fracture size or orientation. Two log-standard deviations have been investigated: $\boldsymbol{\sigma_{log\,T}}$=1 and 2. The CRF reproduces the natural heterogeneities within the fracture plane (30); it is similar to the one used for the IPPA model (see the corresponding paragraph), but with a shorter correlation length of 3 m.
4. **TSL&CRF($\boldsymbol{s}$):** $T_f(x) = T_f * CRF(x, s); T_f = f(\sigma_\infty, l)$ - the transmissivity varies in the fracture plane with a geometric mean T_f that varies in the network from fracture to fracture according to $f(\sigma_\infty, l)$ which is similar to case 2; $CRF(x, s)$ is similar to case 3.

- **Flow channeling indicator**

The flow channeling indicator is defined as the ratio between the total fracture surface per unit volume p_{32} and the flow surface per unit volume d_q calculated from the participation ratio of the flow:

$$d_q = \frac{1}{V}\frac{\left(\int_S qdS\right)^2}{\int_S q^2 dS}$$

where q is the specific discharge at any point of the fracture network and $\boldsymbol{S}$ the fracture surface

S3. List of numerical simulations

Table S2. List of run characteristics

structure	transmissivity	seed	$l_{\min}$	L	p	p_{32}	K	d_q	p_{32}/d_q
constant *p*=3	T=1	seed_0	1	80	3.04	1.449	0.00031	0.198	7.3
constant *p*=8	T=1	seed_0	1	80	7.96	4.926	0.018	3.008	1.6
power law *a*=4	T=1	seed_0	2	150	10.05	0.695	0.10711	0.304	2.3
power law *a*=3.5	T=1	seed_0	2	150	10.07	0.311	0.04711	0.124	2.5
power law *a*=3	T=1	seed_0	2	150	10.04	0.156	0.02759	0.07	2.2
open genetic	T=1	seed_10	2	150	14.54	0.433	0.040	0.142	3.0
open genetic	T=1	seed_3	2	150	14.75	0.437	0.050	0.150	2.9
open genetic	T=1	seed_5	2	150	15.54	0.446	0.045	0.142	3.1
open genetic	T=1	seed_7	2	150	14.90	0.421	0.030	0.130	3.2
open genetic	T=1	seed_9	2	150	17.20	0.436	0.040	0.140	3.1
open genetic	T=1 CRF(1)	seed_5	2	150	15.54	0.446	0.04163	0.108	4.1
open genetic	T=1 CRF(2)	seed_5	2	150	15.54	0.446	0.0332	0.063	7.1
open genetic	TSL	seed_10	2	150	14.54	0.433	0.001	0.046	9.4
open genetic	TSL	seed_3	2	150	14.75	0.437	0.005	0.036	12.2
open genetic	TSL	seed_5	2	150	15.54	0.446	0.002	0.053	8.4
open genetic	TSL	seed_9	2	150	17.20	0.436	0.003	0.049	8.9
open genetic	TSL CRF(1)	seed_5	2	150	15.54	0.446	0.0018	0.038	11.7
open genetic	TSL.CRF(2)	seed_5	2	150	15.54	0.446	0.00144	0.021	21.0
ippa	T=1	seed_10	2	150	10.25	0.171	0.002	0.017	10.0
ippa	T=1	seed_3	2	150	8.10	0.163	0.007	0.020	8.1
ippa	T=1	seed_5	2	150	9.30	0.173	0.0049	0.020	8.6
ippa	T=1	seed_7	2	150	8.90	0.163	0.001	0.014	11.9
ippa	T=1	seed_9	2	150	11.00	0.173	0.009	0.027	6.4
ippa	TSL	seed_10	2	150	10.25	0.171	0.000	0.013	12.9
ippa	TSL	seed_3	2	150	8.10	0.163	0.001	0.014	11.8
ippa	TSL	seed_7	2	150	8.90	0.163	0.000	0.010	15.6
ippa	TSL	seed_9	2	150	11.00	0.173	0.001	0.016	11.0

S4. Eulerian vs Lagrangian velocity distributions

In Figure S1a,b we show examples of Eulerian and Lagrangian inverse velocity distributions (n_L and n_E, respectively). In Figure S1c we plot the tail power law exponents (Eulerian δ_E and Lagrangian δ_L) in the range where the power law trend is established, over the same velocity range for both distributions. In Figure S1d, we plot the channeling factor for the different models of structure and transmissivity.

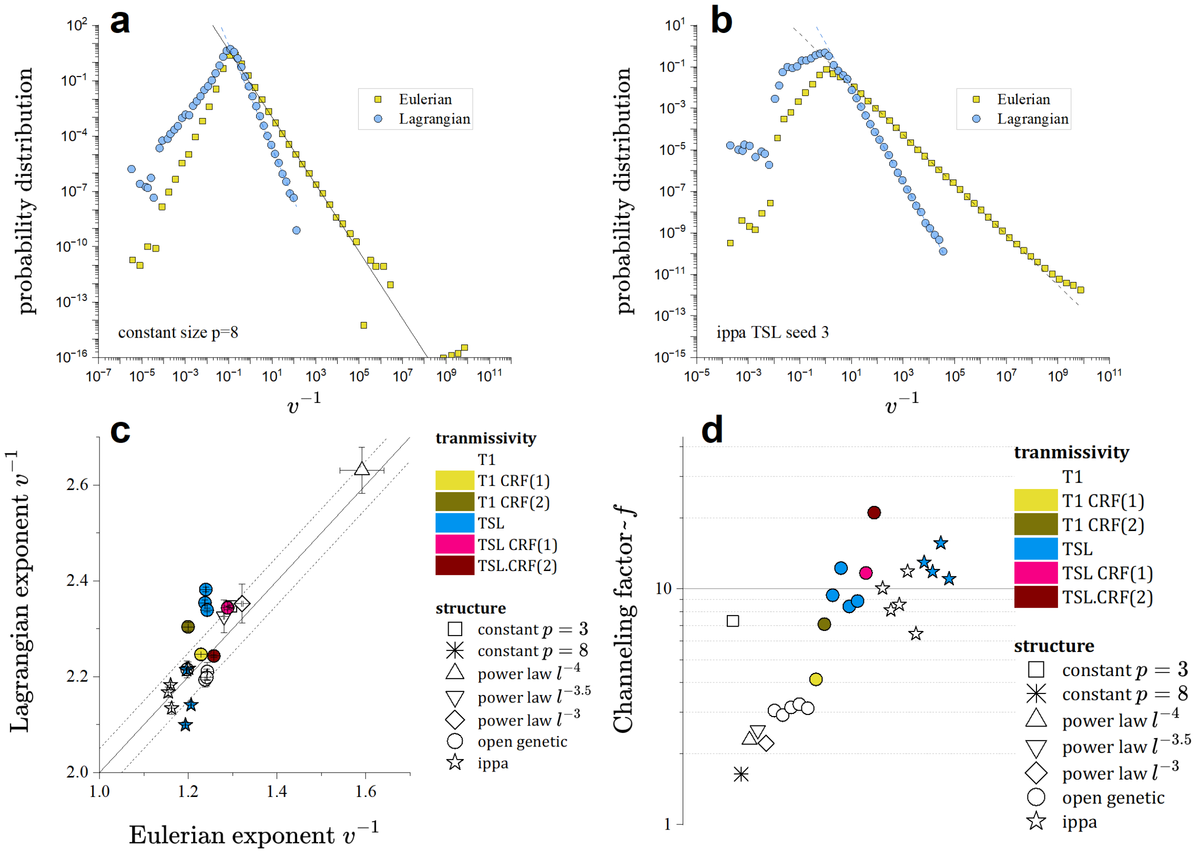

Figure S1. *a*) and *b*) Eulerian and Lagrangian distributions, n_L and n_E, respectively, for two DFNs from the most simple model (constant size with $T = 1$ in **a**) to the one of the most complex model (IPPA with variable transmissivity TSL in **b**). The power law fits are shown for each of the curves. **c**) Comparison between the exponents of the long-tail fits of the v^{-1} distributions δ_E and δ_L measured respectively from the Eulerian maps (horizontal axis) or from the Lagrangian sampling of the particles (vertical axis). **d**) Channeling factor for the different models of structure and transmissivity. The x axis is the model number. The shape of the symbol indicates the DFN structure model, and the color indicates the transmissivity model (see the legend on the right).

S5. Evolution of breakthrough curves and transport moment with travel distance

We investigate how the proposed BTC model parameters are sensitive to the structure and transmissivity DFN models defined in the model database, and to the distance between injection and recovery location.

To illustrate the transport dynamics in DFN simulations, we consider two extreme cases. The first (**Figure S2**, left column) corresponds to a simple DFN with constant fracture size and transmissivity, and a percolation parameter of 8, well above the percolation threshold. This model is a good candidate for obeying the macrodispersion equation at long distance. The second simulation (**Figure S2**, right) corresponds to an open genetic model with a constant transmissivity, which is much more heterogeneous than the first case.

For the constant fracture size model (**Figure S2**, left column), the two-regime fit appears to hold for the BTC. The mean and median are approximately the same and vary linearly with the distance from the inlet side. In contrast, the standard deviation is much smaller than the mean and varies less than linearly with distance (~$x^{0.6}$), close to the prediction of the central limit theorem in $x^{0.5}$ (**Figure S2**, middle row). This is consistent with a long-tail exponent a greater than 3 (a~4), which is not stable upon summation

(**Figure S2**, bottom row). Visually, the distribution is very peaked compared to the others, which is confirmed by the large exponents s and k of the SIG, the latter increasing continuously with distance without a limit (**Figure S2**, bottom row). Note that this run is the exception in our set of runs where one of the exponents converges to a limit at large distance.

For the open genetic model (**Figure S2**, right), the log-log slope of the long-tail power law appears to be approximately constant whatever the distance (**Figure S2**, top row). In contrast to the first case, the mean is larger than the median but smaller than the standard deviation (**Figure S2**, middle row, right column). All three increase linearly with the distance from the inlet side. The transition between the two regimes is constant at a small distance and then increases linearly with distance in a constant ratio to the mean. The three fit exponents tend to be constant at long distances (**Figure S2**, bottom row). The long-tail exponent a is less than 3, consistent with the observation of a large standard deviation. The stretched exponent s is about 1, so the first regime fit is a pure inverse gamma. The exponent k is 5, significantly larger than a, indicating a clear transition in the tail of the distribution.

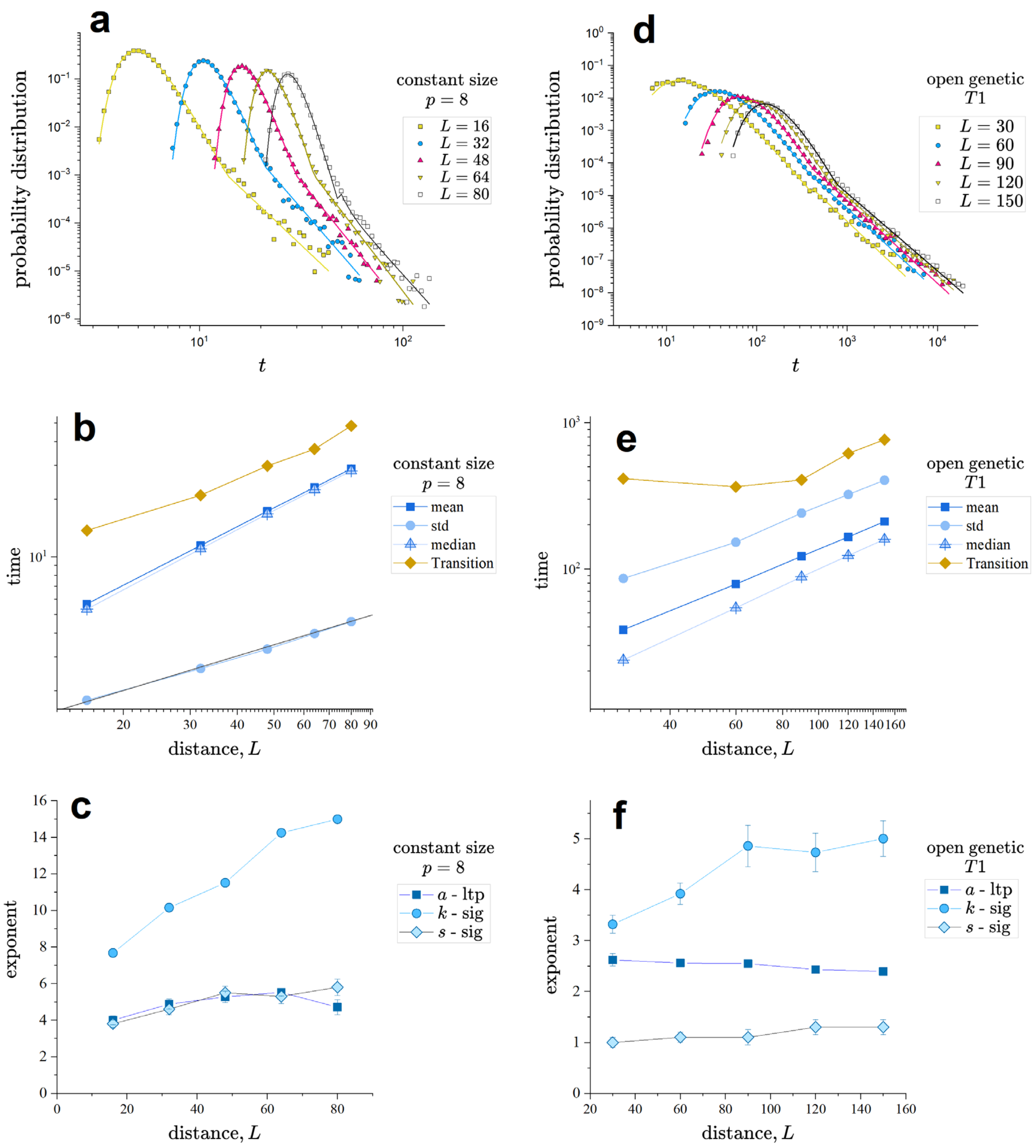

Figure S2. BTC properties from transport simulations for 2 DFN models, with structure and transmissivity model with constant size, constant transmissivity (left column, a, b, c) and open genetic and constant transmissivity (right column, d, e, f) and BTC at successive distances from the inlet side (top row). Middle row, b and e: Evolution of the BTC distribution characteristics (mean, standard deviation, median and transition between the two regimes) with distance to the inlet. Bottom row,c and f: Evolution of the model exponents (, and).

S6. Fit of breakthrough curves

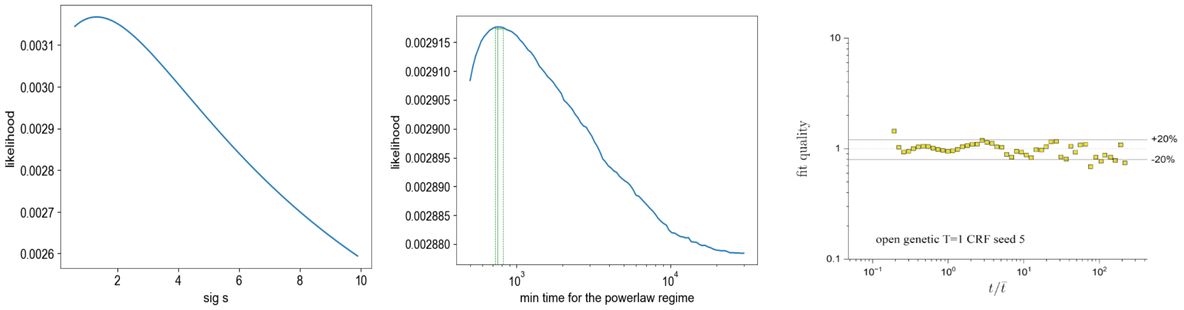

Figure S3. Left: Likelihood of the total fitting function including the stretched inverse gamma and the longtail power law, as a function of the transition between the sig and power-law regimes. Middle: Likelihood of the total fitting function including the stretched inverse gamma and the longtail power law, as a function of the transition between the sig and power-law regimes. Right: Fit quality calculated as the ratio between the BTC and the fit whose parameters are calculated by the three-stage maximum likelihood procedure described in the section

The fit was obtained in several steps:

- We first calculate the stretched inverse gamma for short times ($t < t_o$).

  The (incomplete) stretched inverse gamma is: $sig(t) = \frac{f}{\Gamma_{inc}\left(\frac{k-1}{s};\left(\frac{\beta}{t_o}\right)^s\right)} \frac{s\,\beta^{k-1}}{\Gamma\left(\frac{k-1}{s}\right)} t^{-k} \exp\left(-\left(\frac{\beta}{t}\right)^s\right)$

  Γ and Γ_{inc} are the gamma and lower incomplete gamma functions, respectively, s and k are the exponents of the stretched gamma function, β is a characteristic time (related to the average time and to s) and f is the percentage of transfer times that follow the sig distribution: $\int_0^{t_o} sig(t)dt = f$.

  First, we derive an analytical expression for the parameter k corresponding to the maximum likelihood (ML) of sig for constant s: $k_{ML}(s)$. Then, the likelihood of the sig function is computed as a function of s with $k = k_{ML}(s)$. The ML parameters are s_{ML}, i.e., the value of s corresponding to the maximum likelihood, and $k = k_{ML}(s_{ML})$ (**Figure S3**, left).

- Then, we derive the parameters of the large time power-law for $t > t_1$:

  $$pw(t) = (1-f)\frac{a-1}{t_1}\left(\frac{t}{t_1}\right)^{-a}$$

  t_1 is visually determined as a time for which the power-law fit is valid (i.e., greater than the transition between the two regimes. We compute the exponent a of the long tail power law fit from a maximum likelihood for all values greater than t_1. In practice, the transition is first estimated visually (a quantitative estimate is made in the next step), and t_1 is taken as a time greater than the transition, but not too large to maintain a time range large enough for the power-law fit.

- We derive the transition between both regimes by considering the fit equal to:

  $$fit(t) = sig(t) + pw(t)$$

  The parameter f determines the transition between the two regimes. It is obtained as the maximum likelihood of fit with respect to data (**Figure S3**, middle).

  Note that t_o and t_1 can be either equal to the transition time or greater and less than it, but the final result is not so dependent on the choice of these values.

- Finally, we check the ability of the fit to model the data (figure below). The deviation of the fit from the data is less than 20% (see **Figure S3**, right).

S7. Examples of breakthrough curves

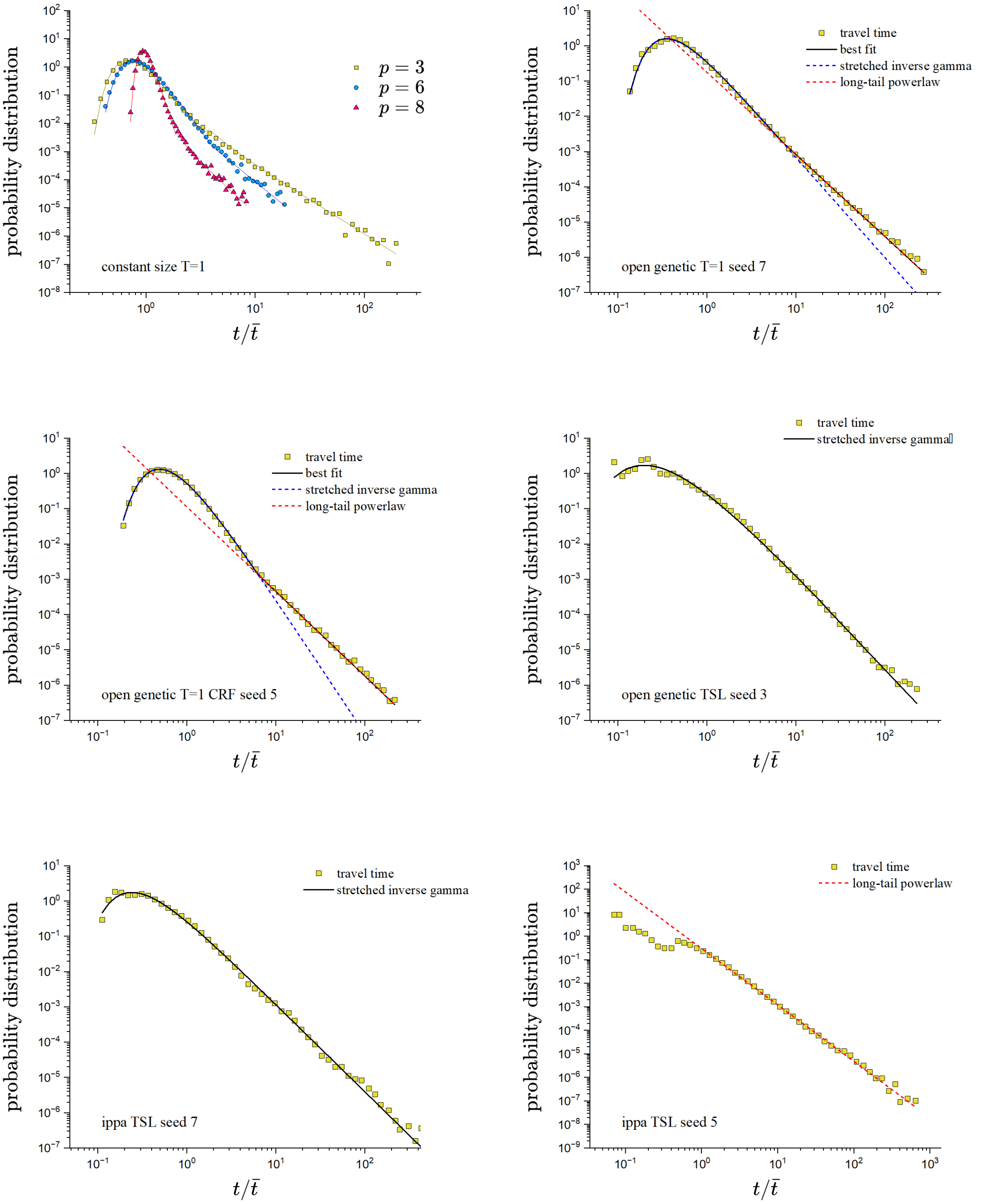

Figure S4. Examples of travel time distributions for different DFN modes, whose name is indicated on each plot.

S8. Synthesis of transport parameters

The three BTC exponents and the two velocity exponents are synthesized in **Figure S5** for all the runs classified from left to right by increasing complexity in the structure (from constant-size networks to DFN with in-plane open patches, see Section 2.2). Most of the long tail exponents $\boldsymbol{a}$ are smaller than 3, indicating that the second moment and the standard deviation of the BTC is controlled by the largest travel times. The only exceptions are the high-density model with constant fracture sizes and a large

percolation parameter of 8, and the power-law size model $\boldsymbol{l}^{-4}$, which is not that different from the constant size model in terms of connectivity (16).

The gamma exponent $\boldsymbol{k}$ varies between 2 and 7 with the two same exceptions as for $\boldsymbol{a}$ that takes values of 10-12. The trend is that the more complex is the structure, the smaller is $\boldsymbol{k}$. The stretched exponent $\boldsymbol{s}$ is around 1 for a large majority of modes, indicating that the BTC is mostly a simple inverse gamma before the transition.

The exponents of the $\boldsymbol{v}^{-1}$ distribution are less variable than the other exponents. The Lagrangian exponents range between 2.1 and 2.4 with the exception of the high-density constant size simulation ($\boldsymbol{p} = \mathbf{8}$). The Eulerian exponent is between 1 and 1.2 for the runs with constant transmissivity ($\boldsymbol{T} = \mathbf{1}$) and slightly larger for the runs with varying transmissivity (TSL) or complex structure (IPPA).

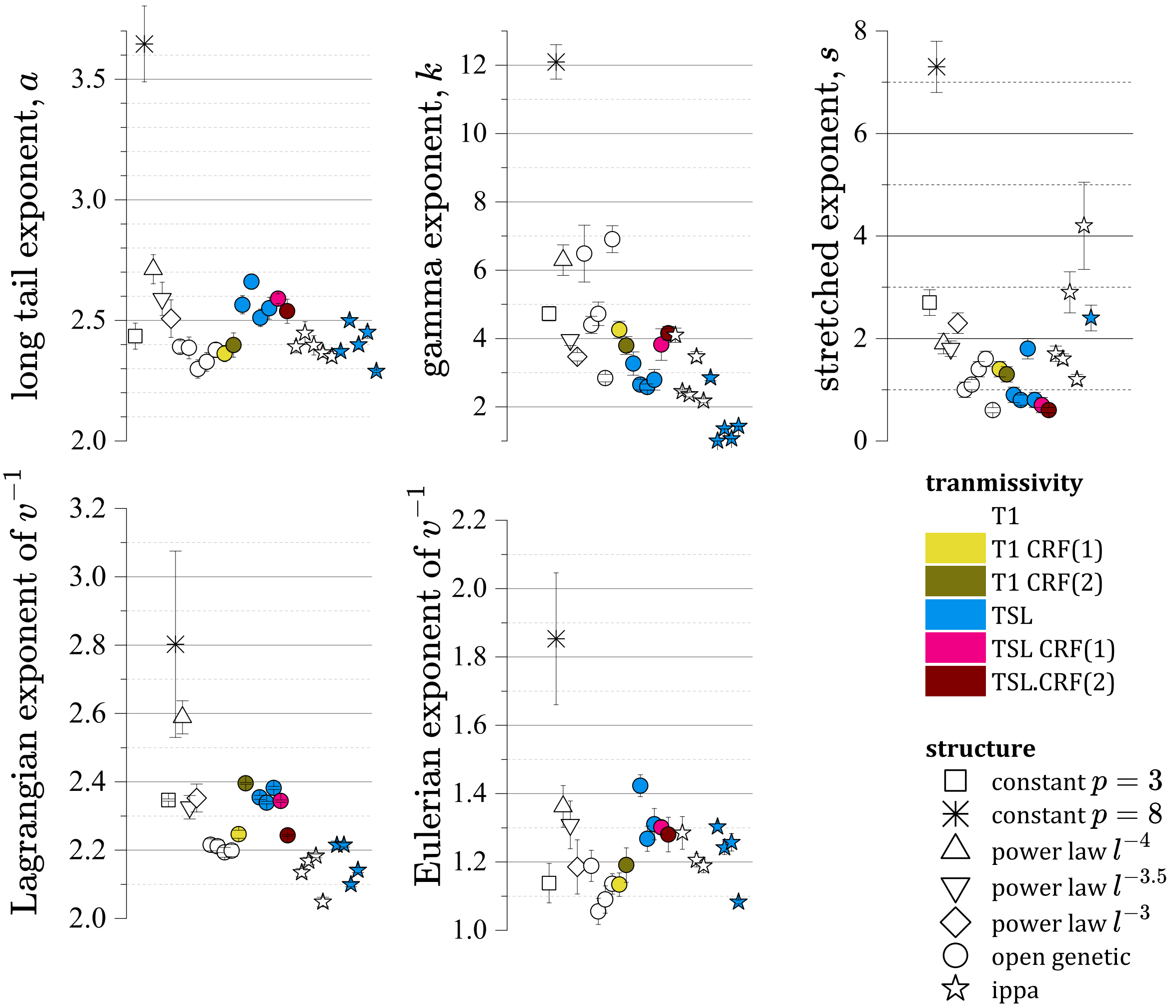

Figure S5. Synthesis of the characteristic law exponents describing the travel time and velocity distributions. Top row: the travel-time exponents; from left to right: the long-tail exponent $\boldsymbol{a}$, the stretched inverse gamma exponent $\boldsymbol{k}$, and the stretched exponent $\boldsymbol{s}$. Bottom row: the exponent of the $\boldsymbol{v}^{-1}$ Lagrangian (left) and Eulerian (right) distribution. The color scale and shape list indicate the transmissivity model and DFN structure, respectively, as shown in the bottom-right legend. For all the graphs, the $\boldsymbol{x}$ axis is the run number (see Table S2-1).

It is noteworthy that the geo-DFN structure is a better control of the exponents (Figure S4-1 and Table S2-1) than the percolation parameter, although the latter controls the network connectivity (15, 16). The three power-law size simulations performed with the same percolation parameter, but different fracture-size exponents illustrate this statement since the resulting exponents, $\boldsymbol{a}$, $\boldsymbol{k}$ or $\boldsymbol{s}$, are strikingly different from one simulation to another (Figure S4-1).

The characteristics of the transition between the two regimes are shown in Figure S4-2. For most of the runs, the transition time (t_T) occurs well above the mean, with the notable exception of the IPPA/TSL models, where the first part of the travel time distribution is not a SIG. For these models, the long-tail power-law regime describes most of the distribution. Figure S4-2 shows also the contribution of the power-law regime to the first (Figure S4-2, middle) and second (Figure S4-2, right) moments of the travel time distribution. There is a strong correlation between the contribution of the power-law regime to these moments and the power-law exponent $\boldsymbol{a}$. When $\boldsymbol{a}$ is less than 3, the long tail power-law regime contributes more than 70% to the second moment of the distribution and thus to the dispersivity. Even the contribution to the first moment (the mean) is not negligible, varying between 10 and 50% depending on the model.

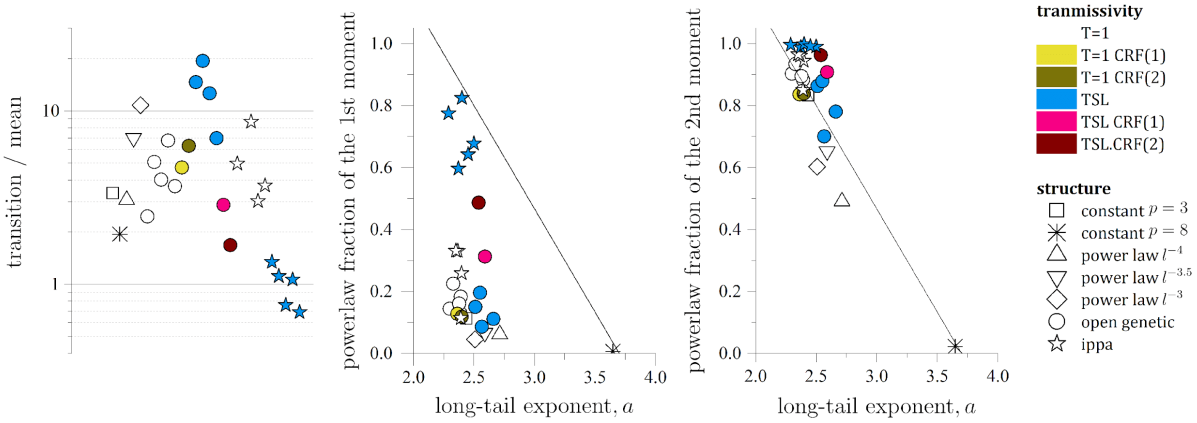

Figure S6. Left, the transition time between the inverse gamma regime and the long-tail power law normalized by the mean travel time presented for all runs in the same way as in Figure S4-1. Middle and right, the long-tail power law contribution to the first and second moments of the travel time distribution, respectively, as a function of the long-tail exponent $\boldsymbol{a}$.

A key difference between the short- and long-term distributions is the difference between the gamma exponent $\boldsymbol{k}$ of the SIG and the long-tail exponent $\boldsymbol{a}$. $\boldsymbol{k} - \boldsymbol{a}$ is an indicator of the kink observed in the figures of the section S4, which marks the change in the BTC regimes. Figure S4-3 shows that the difference between $\boldsymbol{k}$ and $\boldsymbol{a}$ decreases with the variability of the transmissivity model. In the limit when the two exponents are equal, the travel time distribution can be modeled by a single law. This is the case for models with a high variability in transmissivity (open genetic with TSL transmissivity) or with a complex DFN structure (IPPA).

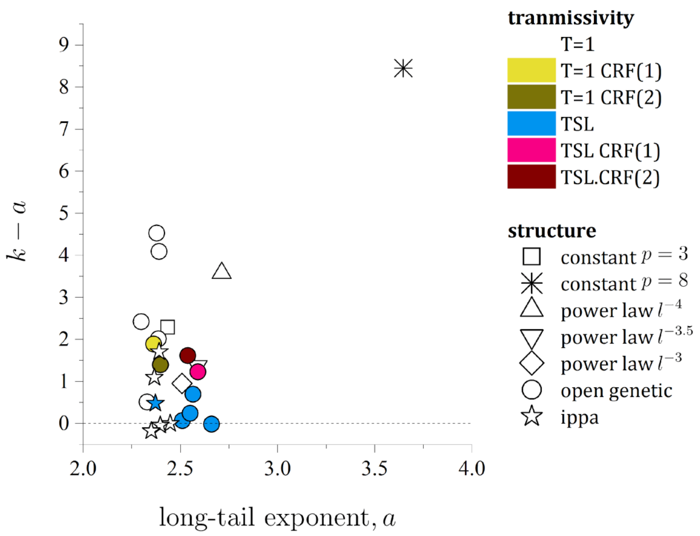

Figure S7. Evolution of the difference between the SIG gamma exponent $\boldsymbol{k}$ and the long-tail exponent $\boldsymbol{a}$ as a function of $\boldsymbol{a}$.

S9. Analysis of "traps"

The traps are defined as the slowest step of each of the streamlines, where each step is defined as the travel path between two intersections. It has been analyzed for an open-genetic model with a TSL transmissivity distribution dependent on fracture orientation and size (blue circle in the previous figures). The trap time ranges from 12% to 100% of the total travel time (TT) with an average at 50% regardless of TT (**Figure S8**, top left). The variability decreases as TT increases, with a tendency for the trap to represent a large percentage of TT. The R^2 correlation coefficient of 0.82 shows the good correlation between trap time and travel time, which is confirmed by the similarity of the two distributions.

By selecting trap times, whose distribution is very similar to that of TT, it is possible to analyze which parameter controls the travel time distribution, whether it is the local head gradient, the path tortuosity, or the fracture transmissivity (**Figure S8**, top left, bottom right, bottom left, respectively). The main determinant is the direct head gradient between intersections, i.e. the head difference divided by the intersection distance. The correlation coefficient is 0.2 for the direct head gradient and up to 0.3 for the along-path head gradient presented in the main text, fig. 4. The second determinant is the trap tortuosity with a regression coefficient of 0.05, 4 times smaller than the direct head gradient. Finally, trap time is not correlated with fracture transmissivity. The general picture is that trap time, and thus travel time, is largely controlled by the head gradient between intersections, with trap tortuosity as an additional element that decreases the head gradient along streamlines.

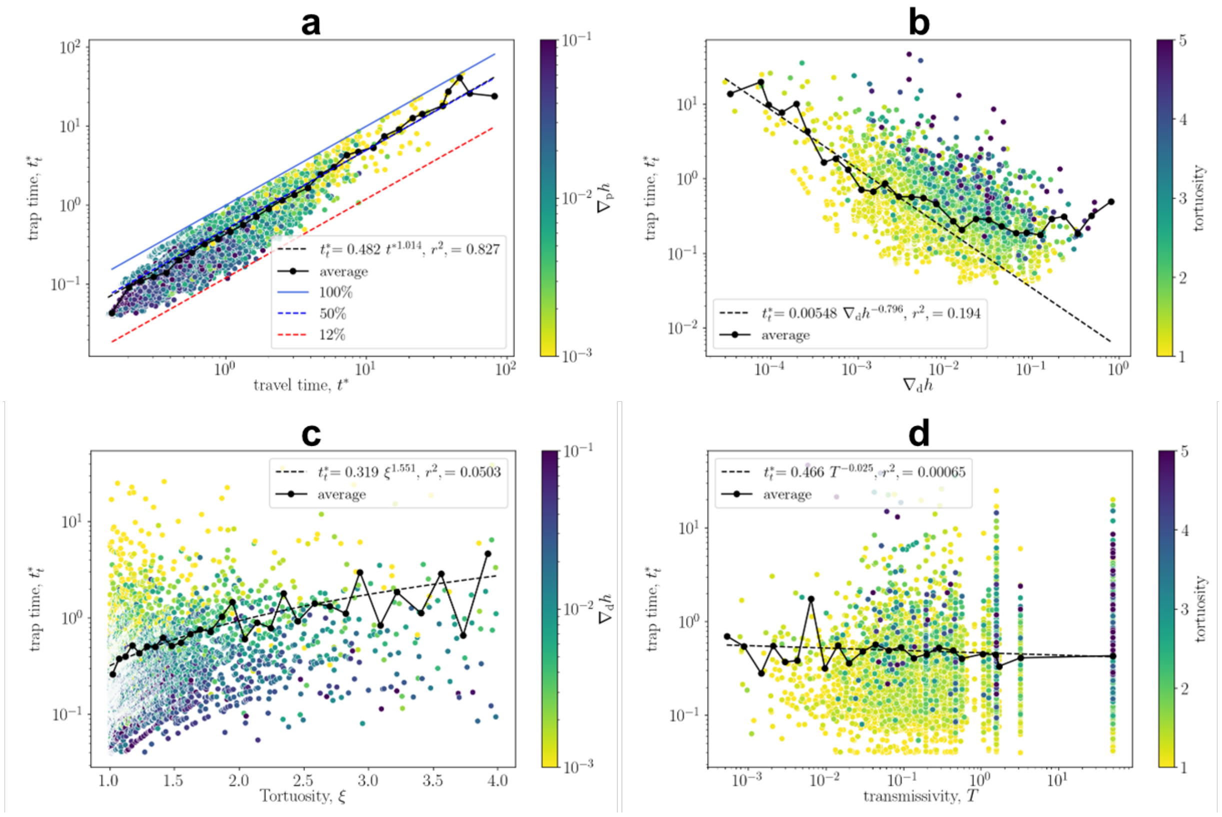

Figure S8. trap time normalized by mean travel time as a function of: *top left*, travel time normalized by its mean; *top right*, direct head gradient between intersections; *bottom left*, fracture transmissivity; and *bottom right*, path tortuosity between intersections. These statistics are derived for an open genetic model with TSL transmissivity distribution.

S10. Classical Continuous Time Random Walk model

The total travel time is assumed to be the sum of time increments Δt resulting from sequential transport events at random velocities of distribution $p(v)$ over structures of fixed characteristic size λ.

eq. 1 $$t = \sum_{i=1}^{n} \Delta t_i$$

with

eq. 2 $$\Delta t_i = \frac{\lambda}{v_i}$$

and

eq. 3 $$p(\Delta t) = p(v)\frac{dv}{d\Delta t} = p(v)\frac{\lambda}{\Delta t^2} \sim p(v^{-1}).$$

A power low distribution of velocities in the low velocity range hence leads to power law statistics of temporal increments, $p(\Delta t) \sim t^{a}$. When velocity statistics follow a power law $p(v^{-1}) \sim (v^{-1})^{-\delta}$, as observed in our simulations (**Figure S1**a), this implies $a = \delta$. For $a \leq 3$, power law statistics are stable upon summation since the sum is dominated by the occurrence of extreme events. Owing to Eq. 3, this leads to power statistics of the total travel time, $p(t) \sim t^{-a}$.

S11. Coupled Continuous Time Random Walk model

To test and validate our conclusions on the impact of velocity dependent correlation length on anomalous dispersion, we simulate the coupled CTRW model. We thus solve the following equations with random walk simulations:

$$t = \sum_{i=1}^{n} \Delta t_i \quad \text{eq. 4}$$

$$x = \sum_{i=1}^{n} \Delta x_i \quad \text{eq. 5}$$

with

$$\Delta t_i = \frac{\Delta x_i}{v_i} \quad \text{eq. 6}$$

and

$$\Delta x_{i_i} = v_i{}^{\mu} \quad \text{eq. 7}$$

where the velocity v_i is sampled from the power law distribution,

$$\mathrm{p}(v_i) \sim v_i{}^{\delta-2} \quad \text{eq. 8}$$

equivalent to

$$\mathrm{p}(v_i{}^{-1}) \sim (v_i{}^{-1})^{-\delta} \quad \text{eq. 9}$$

As discussed in the manuscript, this model predicts a power law decay of large travel times as,

$$\mathrm{p}(t) = t^{-\frac{\delta-\mu}{1-\mu}} \quad \text{eq. 10}$$

This prediction is verified in Fig. S9, demonstrating the stability of this scaling with distance for $\mu < \frac{(3-\delta)}{2}$ and the difference with the scaling predicted by the standard CTRW model, $\mathrm{p}(t) = t^{-\delta}$. The effect of enhanced channeling, described here by increasing the value of the coupling exponent μ, is to reduce the tailing of long travel times, as predicted by Eq. 10. For $\mu > \frac{(3-\delta)}{2}$, reached for $\mu = \frac{1}{2}$ and $\mu = \frac{2}{3}$ for the case of Fig. S9, the stability of the power law tailing is lost and transport transitions to Fickian dispersion.

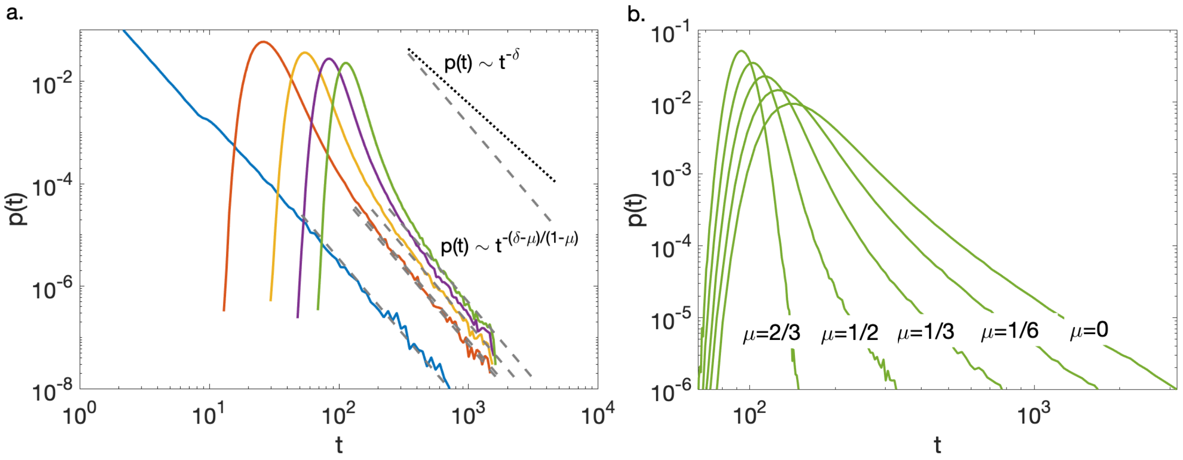

Figure S9. Random walk simulations of the coupled CTRW model. a. Breakthrough curves at different distances x=1,20,40,60,80 and 100 for $\delta = 2.2$ and $\mu = \frac{1}{3}$, similar to the exponents infered from most IPPA simulations. The dashed lines show the theoretical scaling of Eq. 10., while the dotted lines show

the prediction of the standard CTRW model. b. Breakthrough curves at distance x=100 for $\delta = 2.2$ and increasing channeling $\mu = 0, \frac{1}{6}, \frac{1}{3}, \frac{1}{2}, \frac{2}{3}$.

References

1. M. B. Stephens *et al.* (2008) Geology Forsmark. Site descriptive modelling Forsmark stage 2.2. in *SKB Rapport* (Svensk Kärnbränslehantering AB, Stockholm), pp 1-224.
2. I. Olofsson *et al.* (2007) Site descriptive modelling Forsmark, stage 2.2. in *SKB R-Reports* (SKB, Stockhölm).
3. A. Fox, P. La Pointe, J. Hermanson, J. Öhman (2007) Statistical geological discrete fracture network model. Forsmark modelling stage 2.2. in *SKB Rapport* (Svensk Kärnbränslehantering AB, Stockholm), pp 1-271.
4. M. B. Stephens (2010) Forsmark site investigation. Bedrock geology-overview and excursion guide. in *SKB report*, ed SKB (Svensk Kärnbränslehantering AB, Stockholm).
5. J.-O. Selroos *et al.* (2022) Methodology for discrete fracture network modelling of the Forsmark site. Part 1 – concepts, data and interpretation methods. in *SKB reports* (Svensk Kärnbränslehantering AB, Solna (Sweden)), p 257.
6. R. Glamheden *et al.* (2007) Rock mechanics Forsmark. Site descriptive modelling Forsmark stage 2.2. in *SKB reports* (Swedish Nuclear Fuel and Waste Management Co.).
7. S. Follin (2008) Bedrock hydrogeology Forsmark, Site descriptive modelling, SDM-Site Forsmark, R-08-95. in *SKB reports*.
8. S. Follin *et al.*, A methodology to constrain the parameters of a hydrogeological discrete fracture network model for sparsely fractured crystalline rock, exemplified by data from the proposed high-level nuclear waste repository site at Forsmark, Sweden. *Hydrogeol. J.* **22**, 313-331 (2014).
9. A. Öhberg, P. Rouhiainen (2000) Posiva groundwater flow measuring techniques. in *Report POSIVA* (Posiva Oy, Helsinki).
10. S. Follin, M. Stigsson, A transmissivity model for deformation zones in fractured crystalline rock and its possible correlation to in situ stress at the proposed high-level nuclear waste repository site at Forsmark, Sweden. *Hydrogeol. J.* **22**, 299-311 (2014).
11. J. D. Hyman, G. Aldrich, H. Viswanathan, N. Makedonska, S. Karra, Fracture size and transmissivity correlations: Implications for transport simulations in sparse three-dimensional discrete fracture networks following a truncated power law distribution of fracture size. *Water Resour. Res.* **52**, 6472-6489 (2016).
12. V. Cvetkovic, A. Frampton, Solute transport and retention in three-dimensional fracture networks. *Water Resour. Res.* **48** (2012).
13. P. Davy, R. Le Goc, C. Darcel, J.-O. Selroos, Scaling of fractured rock flow. Proposition of indicators for selection of DFN based flow models. *Comptes Rendus. Géoscience* **355**, 1-23 (2023).
14. D. Stauffer, A. Aharony, *Introduction to percolation theory, second edition* (Taylor and Francis, Bristol, 1992).
15. O. Bour, P. Davy, Connectivity of Random Fault Networks Following a Power Law Fault Length Distribution. *Water Resour. Res.* **33**, 1567-1583 (1997).
16. O. Bour, P. Davy, On the Connectivity of Three-Dimensional Fault Networks. *Water Resour. Res.* **34**, 2611-2622 (1998).
17. A. Piggott, Fractal relations for the diameter and trace length of disc-shaped fractures. *Journal of Geophysical Research* **102**, 18121-128126 (1997).
18. C. Darcel, O. Bour, P. Davy, Stereological analysis of fractal fracture networks. *Journal of Geophysical Research* **108** (2003).
19. P. Davy, C. Darcel, O. Bour, R. Munier, J. R. de Dreuzy, A note on the angular correction applied to fracture intensity profiles along drill core. *J. Geophys. Res. B Solid Earth* **111**, n/a-n/a (2006).

20. I. I. Bogdanov, V. V. Mourzenko, J. F. Thovert, P. M. Adler, Effective permeability of fractured porous media with power-law distribution of fracture sizes. *Physical Review E* **76**, 036309 (2007).
21. C. E. Renshaw, Connectivity of joint networks with power law length distributions. *Water Resour. Res.* **35**, 2661 - 2670 (1999).
22. J. C. S. Long, P. A. Witherspoon, The Relationship of the Degree of Interconnection to Permeability in Fracture Networks. *Journal of Geophysical Research* **90**, 3087-3098 (1985).
23. J. Hyman, M. Dentz, A. Hagberg, P. K. Kang, Linking structural and transport properties in three-dimensional fracture networks. *J. Geophys. Res. B Solid Earth* **124**, 1185-1204 (2019).
24. A. Frampton, V. Cvetkovic, Significance of injection modes and heterogeneity on spatial and temporal dispersion of advecting particles in two-dimensional discrete fracture networks. *Adv. Water Resour.* **32**, 649-658 (2009).
25. P. K. Kang, M. Dentz, T. Le Borgne, S. Lee, R. Juanes, Anomalous transport in disordered fracture networks: Spatial Markov model for dispersion with variable injection modes. *Adv. Water Resour.* **106**, 80-94 (2017).
26. M. Dentz, P. K. Kang, A. Comolli, T. Le Borgne, D. R. Lester, Continuous time random walks for the evolution of Lagrangian velocities. *Physical Review Fluids* **1**, 074004 (2016).
27. E. Bonnet *et al.*, Scaling of fracture systems in geological media. *Rev. Geophys.* **39**, 347-383 (2001).
28. J. D. Hyman, M. Dentz, A. Hagberg, P. K. Kang, Emergence of Stable Laws for First Passage Times in Three-Dimensional Random Fracture Networks. *Phys Rev Lett* **123**, 248501 (2019).
29. P. Davy *et al.*, A likely universal model of fracture scaling and its consequence for crustal hydromechanics. *Journal of Geophysical Research* **115**, 1-13 (2010).
30. Y. Meheust, J. Schmittbuhl, Flow enhancement of a rough fracture. *Geophys. Res. Lett.* **27** (2000).
